# Enhanced antitumor efficacy of a novel oncolytic vaccinia virus encoding a fully monoclonal antibody against T-cell immunoglobulin and ITIM domain (TIGIT)

**DOI:** 10.1016/j.ebiom.2021.103240

**Published:** 2021-02-10

**Authors:** Shuguang Zuo, Min Wei, Bohao He, Anxian Chen, Shiqun Wang, Lingkai Kong, Yenan Zhang, Gang Meng, Tiancheng Xu, Jingyi Wu, Fuming Yang, Hailin Zhang, Shibing Wang, Ciliang Guo, Junhua Wu, Jie Dong, Jiwu Wei

**Affiliations:** Jiangsu Key Laboratory of Molecular Medicine, Medical School of Nanjing University, No. 22, Hankou Road, Nanjing 210093, China

**Keywords:** Oncolytic virus, Vaccinia virus, Checkpoint inhibitors, TIGIT, Cancer immunotherapy

## Abstract

**Background:**

Oncolytic virotherapy with vaccinia virus (VV) can lead to effective anti-tumor immunity by turning “cold” tumors into “hot” tumors. However, its therapeutic potential is affected by the tumor's local immunosuppressive tumor microenvironment (TME). Therefore, it is necessary to explore the use of immune checkpoint inhibitors to arm oncolytic VVs to enhance their anti-tumor efficacy.

**Methods:**

A novel recombinant oncolytic VV, VV-α-TIGIT, which encoded a fully monoclonal antibody against T-cell immunoglobulin and ITIM domain (TIGIT) was generated by homologous recombination with a shuttle plasmid. The anti-tumor efficacy of the VV-α-TIGIT was investigated in several subcutaneous and ascites tumor models.

**Findings:**

The functional α-TIGIT was sufficiently produced and secreted by tumor cells infected with VV-α-TIGIT, which effectively replicated in tumor cells leading to significant oncolysis. Intratumoral injection of VV-α-TIGIT improved anti-tumor efficacy in several murine subcutaneous tumor models compared to VV-Control (without α-TIGIT insertion). Intraperitoneal injection of VV-α-TIGIT achieved approximately 70% of complete tumor regression in an ascites tumor model. At the same time, treatment with VV-α-TIGIT significantly increased the recruitment and activation of T cells in TME. Moreover, the in vivo anti-tumor activity of VV-α-TIGIT was largely dependent on CD8^+^ T cell-mediated immunity. Finally, the tumor-bearing mice cured of VV-α-TIGIT treatment resisted rechallenge with the same tumor cells, suggesting a long-term persistence of tumor-specific immunological memory.

**Interpretation:**

The recombinant oncolytic virus VV-α-TIGIT successfully combines the advantages of oncolytic virotherapy and intratumorally expression of immune checkpoint inhibitor against TIGIT. This novel strategy can provide information on the optimal design of novel antibody-armed oncolytic viruses for cancer immunotherapy.

**Funding:**

This work was supported by the National Natural Science Foundation of China (81773255, 81472820, and 81700037), the Science and Technology Innovation Foundation of Nanjing University (14913414), and the Natural Science Foundation of Jiangsu Province of China (BK20171098).

**Research in context**

**Evidence before this study**

Oncolytic virotherapy and immune checkpoint inhibitors represent two promising immunotherapy strategies in the field of cancer therapy. It has been previously demonstrated that the intratumoral injection of an engineered oncolytic vaccinia virus (VV) co-expressing the murine GM-CSF and a PD-L1 inhibitor overcomes PD-L1-mediated immunosuppression, leading to the elimination of virus-injected and distant tumors. In addition, it has been shown that the immune checkpoint inhibitor against TIGIT significantly improved the antitumor efficacy of an oncolytic HSV encoding a single-chain variable fragment (scFv) against PD-1. However, there are still no reports that investigate the anti-tumor efficacy of the OVs modified with TIGIT inhibitors.

**Added value of this study**

In the present study, we demonstrated for the first time that VV engineered with an anti-TIGIT monoclonal antibody (α-TIGIT) was an effective strategy for oncolytic immunotherapy by combining viral oncolysis and intratumoral expression of α-TIGIT. Our results indicated that the α-TIGIT armed VV had improved anti-tumor efficacy in several murine tumor models compared to the control VV without α-TIGIT transgene. We also demonstrate that the α-TIGIT armed VV significantly increased the recruitment and activation of CD8^+^ T cells and established long-term tumor-specific immunological memory.

**Implications of all the available evidence**

This study provides a rationale for local delivery of checkpoint inhibitors via an oncolytic virus to overcome the barriers associated with immunologically “cold” tumors. This novel strategy can be extended to other immune checkpoint inhibitors and can provide information on the optimal design of novel antibody-armed oncolytic viruses for cancer immunotherapy.

Alt-text: Unlabelled box

## 1 Introduction

Oncolytic viruses (OVs) are a class of native or recombinant viruses that preferentially infect and replicate in neoplastic cells over normal cells, causing cell lysis [1]. Numerous OVs including herpes simplex virus (HSV), vaccinia virus (VV), adenovirus (Ad), measles virus (MV), vesicular stomatitis virus (VSV), Newcastle disease virus (NDV), and reovirus have been clinically tested [2]. In addition to direct oncolysis, OVs can also indirectly induce innate and adaptive antitumor immunity, which can boost the body's power to recognize, control, and kill cancer cells [3]. Thus, OVs represents another novel and promising immunotherapeutic strategy for the treatment of cancer. OVs can lead to effective infiltration of immune cells, converting a “cold” tumor microenvironment (TME) with few immune cells into a “hot” TME with increased immune cells [4]. Infiltrating immune cells can clear the virus and along with its oncolytic effects. However, activated immune effector cells (such as T cells) can also effectively kill tumor cells and form immunological memory, which can cause a long-term anti-tumor effect [3]. With the activation of T cells, the immunosuppressive checkpoint molecules on the surface of T cells [such as programmed cell death 1 (PD-1), CTL antigen-4 (CTLA-4), and T-cell immunoglobulin and ITIM domain (TIGIT)] will be activated at the same time, which can evade the oncolytic virus activated immune responses.

To solve this problem, a combination of OVs and immune checkpoint inhibitors (ICIs) was used to overcome the immunosuppressive of the TME. A preclinical study demonstrated that in colon and ovarian cancer models an oncolytic VV synergizes with PD-L1 antibody treatment, leading to reduced tumor burden and improved survival of tumor-bearing mice [5]. Besides, the use of the super-agonist IL-15-armed oncolytic virus in combination with PD-1 blockade leads to significant tumor regression and prolongs the survival of mice bearing ovarian or colon cancers [6]. Due to the preclinical success of this combination therapy, many ongoing clinical trials combine OVs and ICIs. Recently, a phase Ib clinical study demonstrated that the combination of talimogene laherparepvec [T-VEC, a human granulocyte-macrophage colony-stimulating factor (GM-CSF)-encoding oncolytic herpes simplex virus 1 (HSV-1)] and pembrolizumab (Keytruda, the first FDA-approved PD-1 inhibitor) has greater efficacy in the treatment of melanoma than either therapy alone [7]. Another phase Ib/II study showed that talimogene laherparepvec plus ipilimumab [a cytotoxic T-lymphocyte antigen-4 (CTLA-4) inhibitor] appeared to have greater efficacy than either T-VEC or ipilimumab monotherapy [8]. However, this combination therapy strategy raises the concern of increased the medical costs of patients. To address these issues, the researchers have designed alternative strategies to enhance the antitumor immunity of oncolytic virotherapy, such as engineering OVs expressing PD-1/PD-L1 inhibitor [[9], [10], [11], [12], [13]].

TIGIT, also called V-set and transmembrane domain-containing protein 3 (Vstm3), V-set and immunoglobulin domain-containing protein 9 (VSIG9), or Washington University cell adhesion molecule (WUCAM), is one of the most promising immune-checkpoint targets to be investigated currently [14]. It is expressed restrictedly on lymphocytes and is more highly expressed on activated natural killer (NK), T, and regulatory T (Treg) cells. Two major ligands for TIGIT are poliovirus receptor (PVR, CD155) and poliovirus receptor-related 2 (PVRL2, CD112, nectin-2), which are expressed by tumor cells as well as myeloid cells. DNAX accessory molecule-1 (DNAM-1, CD226) and CD96 (Tactile) are alternative receptors for PVR and are both expressed on naïve and effector T cells. TIGIT and CD96 interact with PVR to induce immunoinhibitory signals, whereas DNAM-1 delivers a costimulatory signal. Thus, blocking PVR/TIGIT signaling might provide a potential supplement for existing immune checkpoint-based antitumor immunotherapies. A recent study has shown that the TIGIT blockade significantly improved the antitumor efficacy of an oncolytic HSV encoding a single-chain variable fragment (scFv) against PD-1 [15]. However, there are still no reports that investigate the anti-tumor efficacy of the OVs modified with TIGIT inhibitors. In the present study, we engineered a novel oncolytic VV (VV-α-TIGIT) encoding a fully anti-TIGIT monoclonal antibody (mAB). We found that VV-α-TIGIT showed enhanced anti-tumor immunity in several murine tumor models compared to control VV. The engineered oncolytic VV with mABs targeting immune checkpoint is an effective strategy for oncolytic immunotherapy by combining viral oncolysis and intratumorally expression of immune checkpoint antibodies.

## 2 Methods

### 2.1 Cell lines

The cell lines used in this study included HEK293 (human embryonic kidney cell), 4T1 (mouse mammary carcinoma cell), CT26 (mouse colon carcinoma cell), MC38 (mouse colon carcinoma cell), H22 (mouse hepatocellular carcinoma cell), and Hela-S3 (Human cervix carcinoma cell). HEK293 (Cat# CRL-1573, RRID: CVCL_0045), 4T1 (Cat# CRL-2539, RRID: CVCL_0125), CT26 (Cat# CRL-2638, RRID: CVCL_7256), and Hela-S3 (Cat# CCL-2.2, RRID: CVCL_0058) cells were obtained from the American Type Culture Collection (ATCC; USA). MC38 cells (RRID: CVCL_B288) were obtained from the National Cancer Institute (NCI; USA). H22 cells (ID: 3111C0001CCC000309) were obtained from the China Center for Type Culture Collection (CCTCC; Wuhan, China; http://cellresource.cn). HEK293, 4T1, CT26, MC38, and Hela-S3 cells were cultured in Dulbecco's modified Eagle's medium (DMEM; Cat# 11965092, Gibco-Thermo Fisher Scientific, USA) supplemented with 10% fetal bovine serum (FBS; Cat#16000044, Gibco). H22 cells were cultured in RPMI 1640 medium (Cat# 11875093, Gibco) supplemented with 10% FBS (Cat#16000044, Gibco). Hela-S3 cells were cultured in suspension in serum-free medium (Cat# H740KJ, Basalmedia, Shanghai, China) in spinner flasks (Cat# TCB002002, Jetbiofil, Guangzhou, China). All cells were incubated at 37 °C with 5% CO_2_ atmosphere.

### 2.2 Recombinant VV construction, purification, and expansion

The shuttle plasmid pVV-Control was synthesized by GenScript (Nanjing, China). In this plasmid, the reporter gene EGFP and the screening gene guanine-hypoxanthine phosphoribosyl transferase (GPT) are linked by a T2A peptide sequence and are under the control of the p-7.5k early/later promoter. There is another synthesized p-se/l promoter back-to-back with the p-7.5k promoter to control the expression of foreign genes. These chimeric genes are flanked by the left (TK-L) and right (TK-R) fragments of the VV thymidine kinase (TK) gene (**Fig. S1A**). Sequences coding for the hamster anti-mouse TIGIT monoclonal antibody (mAB; Clone 10a7) referred to patent US20090258013A1 which disclosed the sequence of the variable domain of the heavy chain (VH) and the light chain (VL). The VH and VL sequence was linked to the constant region of the heavy chain constant region (GenBank: AF466698.1) and the light chain constant region (GenBank: LC522515.1) of the mouse IgG2a to form a complete heavy chain (HC) and light chain (LC), respectively. Then, a secreted Lucia gene fragment was linked to the heavy chain gene fragment, and the latter is linked to the light chain gene fragment via an E2A peptide sequence to form a Lucia-HC-2A-LC fragment. Finally, the Lucia-HC-2A-LC fragment was synthesized by GenScript and subclone into the pVV-Control plasmid to construct a recombinant shuttle plasmid (pVV-α-TIGIT) for expressing the α-TIGIT mAB under the control of the p-se/l promoter (**Fig. S1B**).

To generate recombinant oncolytic VVs, a Western Reserve (WR) strain (ATCC VR-1354) was used as a parental virus for homologous recombination. In brief, HEK293 cells were infected with WR at a multiplicity of infection (MOI) of 1 for 2 h and then transfected with the corresponding shuttle plasmid using jetPEI transfection reagent (Cat# 101-10N, Polyplus-transfection, France). The cell extraction solution was used to infect the HEK293 cells in the presence of 25 μg/ml mycophenolic acid (MPA; Cat# A600640, Sangon Biotech, Shanghai, China), 250 μg/ml xanthine (Cat# A601197, Sangon Biotech), and 15 μg/ml hypoxanthine (Cat# A500336, Sangon Biotech). After three cycles of screening, EGFP-positive plaques were isolated, resuspended, and further infect HEK293 cells for two cycles of plaque purification (**Fig. S2A**). After completing the first and second rounds of plaque purification, the following primers were used to amplify the target gene and the viral TK gene to identify whether the recombinant virus was adulterated with the parental vaccinia virus. Primer of target gene: 5′-caggtgatctgtttttattgtggag-3′, 5′-gatctacttccttaccgtgc-3′; Primer of TK: 5′-tgtgaagacgataaattaatgatc-3′, 5′-gtttgccatacgctcacag-3′ (**Fig. S2B**). Recombinant vaccinia virus successfully screened by plaque purification was further expanded by Hela-S3 cells in 6-well plates, cell culture dishes, and cell culture spinner flasks (**Fig. S2A**).

### 2.3 Luciferase assay

Since the α-TIGIT antibody encoded by the VV-α-TIGIT virus is fused with a luciferase reporter molecule, the antibody can be quantified by detecting the luciferase activity. Hela-S3 cells were seeded in 6-well plates at 5 × 10^5^ cells per well. Cells were grown to more than 90% confluence and were infected with recombinant VV at MOI of 1. After 24 h, both cells and supernatants were collected and centrifuged to separate them. After removing the supernatants, the cell pellets were resuspended in the same amount of DMEM media as the supernatants, and the cells were lysed by repeated freezing and thawing to release the luciferase. Then, 10 μl of QUANTI-Luc™ substrate (Cat# rep-qlc1, InvivoGen) was added to 100 μl of the cell lysate and the supernatants to detect the luciferase activity. The secreted α-TIGIT in the supernatants of VV-infected 4T1, CT26, MC38, and H22 cells was quantified similarly as in Hela-S3 cells. For detection of in vivo α-TIGIT expression, blood and tumor tissues were collected from mice on day 3 after VV-treatment. Then, 100 mg of tumor sample was added with 200 μl PBS and lysed with a homogenizer. After that, the supernatants and sera were separated by centrifugation. A similar luciferase assay was performed to quantify the levels of the α-TIGIT.

To detect whether the secreted α-TIGIT antibody could bind to the recombinant TIGIT protein (r-TIGIT; Cat# 50939-M38H, Sino Biological, Beijing, China) of the mouse, a luciferase-linked immunosorbent assay was performed. Briefly, 96-well plates were coated with r-TIGIT at a concentration of 10 μg/ml. Then, supernatants of VV-infected Hela-S3 cells were collected and added to the coated wells and incubated at 4 °C for 12 h. After that, the cell culture supernatant was removed, washed 3 times with 1 × TBS buffer, and then 50 μl of QUANTI-Luc™ substrate was added into the wells to detect the luciferase activity.

### 2.4 MTT and CCK8 assay

For the MTT assay, 4T1, CT26, and MC38 cells were seeded in a 96-well plate at 5 × 10^3^ cells per well and were incubated at 37 °C with 5% CO_2_ atmosphere. After 90% confluence, cells were infected with recombinant oncolytic VVs at indicated MOIs in triplicate. After a 48- or 72-h incubation, 20 μl of MTT solution (5 mg/ml; Cat# IM0280, Solarbio, Beijing, China) was added to each well and the cells were continuously incubated for 4 h. After the incubation, the supernatants were removed from the wells and 150 μL of isopropanol was added to each well to solubilize the formazan crystals. The absorbance (A) was measured at 570 nanometers using a microplate reader (SpectraMax M3, Molecular Devices, USA). The cell viability is calculated according to the following formula: cell viability (%) = (A_treatment_ − A_blank_)/(A_control_ − A_blank_) × 100%. For the CCK8 assay, H22 cells were seeded in a 96-well plate at 1 × 10^4^ cells per well and were infected with VVs at indicated MOIs in triplicate. After a 48- or 72-h incubation, 10 μl of CCK-8 solution (Cat# C0037, Beyotime, Shanghai, China) was added to each well of the plate and incubated for 1 h. The absorbance was measured at 450 nm using a microplate reader. The cell viability is calculated similarly to the MTT assay.

### 2.5 Crystal violet staining

Tumor cells were plated and infected with recombinant oncolytic VVs similar to the MTT assay. After 72 h of incubation, the medium was removed and the crystal violet solution (Cat# C0121, Beyotime, Shanghai, China) was added to the wells and incubated for 5 min. After incubation, the crystal violet solution was removed from the wells and washed 5 times with dd H_2_O. The image was acquired using a scanner.

### 2.6 Replication of the oncolytic VVs in tumor cells

Tumor cells were seeded in a 24-well plate at 5 × 10^4^ cells per well and placed in a 5% CO_2_ incubator at 37 °C. After 90% confluence, cells were infected with recombinant oncolytic VVs at MOIs of 0.1. The cells were harvested after 24, 48, 72, and 96 h and then disrupted by three freeze-thaw cycles, and centrifuged at 3000 g for 10 min. The virus supernatants were collected and the virus titer was determined by a TCID50 method.

### 2.7 Animal experiments

Six-week-old male C57BL/6 and male or female BALB/c mice were purchased from the Model Animal Research Center of Nanjing University (Nanjing, China). All animal experiments were performed following the guidelines that had been approved by the Animal Care and Use Committee of the Medical School of Nanjing University. In the subcutaneous (S.C.) tumor model, 4T1, CT26, MC38, or H22 tumor cells were injected subcutaneously into the right flank of the mice. When the tumor reached 50 mm^3^, the mice were randomly divided into different groups and treated with intratumoral (I.T.) injection of PBS or recombinant VVs. In the hepatocellular carcinoma ascites tumor model, H22 cells were implanted intraperitoneally (I.P.) into mice. When ascites formed, the mice were randomly divided into different groups and treated with an intraperitoneal injection of PBS or recombinant VVs. Tumor length (L) and width (W) were measured every two days using Vernier calipers and the tumor size (V) was calculated by the following formula: V = (L×W^2^)/2. For the CD8^+^ T or NK cell depletion experiments, C57BL/6 mice were intraperitoneally injected with 500 ug anti-mouse CD8α InVivoMAb (Clone YTS 169.4, Cat# BP0117, BioXCell, USA) or anti-mouse NK1.1 InVivoMAb (Clone PK136, Cat# BP0036, BioXCell).

### 2.8 Flow cytometry

For PVR/CD155 and PD-L1 staining, the cultured 4T1, CT26, MC38, and H22 cells were harvested and stained with monoclonal antibodies against mouse PVR/CD155 (PE; Clone TX56, Cat# 131507, Biolegend, USA) or PD-L1 (PE; Clone 10F.9G2, Cat# 124308, Biolegend, USA) respectively for 15 min at room temperature and immediately analyzed with a FACS Caliber cytometer (BD Biosciences, USA).

Ascites fluid was collected from the peritoneal cavity of the mice. After counting the cells, 2×10^7^ single-cell suspensions were stained with monoclonal antibodies against mouse CD45 (APC; Clone 30-F11, Cat# 559864, BD), CD3 (FITC; Clone 17A2, Cat# 100204 or PE; Clone 17A2, Cat# 100206, Biolegend, USA), CD8 (PerCP-Cy5.5; Clone 53-6.7, Cat# 551162, BD), CD49b (PerCP-Cy5.5; Clone HMα2, Cat# 103520, Biolegend), NK1.1 (FITC; Clone PK136, Cat# 11-5941-82, Ebioscience, USA) and TIGIT (PE; Clone 1G9, Cat# 142104, Biolegend) for 15 min at room temperature. Cells were fixed with 4% paraformaldehyde (PFA; Cat# 1004965000, Sigma-Aldrich, Germany) and analyzed with a FACS Caliber cytometer (BD). Data analyses were performed with FlowJo software (Treestar, USA).

### 2.9 Enzyme-linked immunosorbent assay (ELISA)

Ascites fluid was collected from the peritoneal cavity of the mice. Then, samples were centrifuged at 600 g for 5 min at room temperature and the supernatants were collected. Mouse IFN-γ in the supernatants was quantified using the ELISA MAX™ Standard Set (Cat# 430801, Biolegend, USA) according to the manufacturing protocol.

### 2.10 Quantitative polymerase chain reaction (qPCR)

Total RNA was extracted with TRIzol reagent (Cat# 15596026, Invitrogen-Thermo Fisher Scientific, USA) and reverse transcribed into cDNA using HiScript III 1st Strand cDNA Synthesis Kit (+gDNA wiper) (Cat# R312, Vazyme, Nanjing, China). The cDNA was amplificated utilizing the AceQ Universal SYBR qPCR Master Mix (Cat# Q511, Vazyme) on a ViiA^TM^ 7 Real-Time PCR System (Applied Biosystems-Thermo Fisher Scientific, USA). The primer sequences used are shown as follows: GAPDH (TCTCCTGCGACTTCAACA, TGTAGCCGTATTCATTGTCA) IFN-γ (CGACACAGTAGAGTGTCGCATG, GAAGTCAAGGTGGAGTGGAGGT), IL-2 (GCGGCATGTTCTGGATTTGACTC, CCACCACAGTTGCTGACTCATC), IL-6 (CCACCAAGAACGATAGTCA, TTGTCACCAGCATCAGTC), and IL10 (CAAACAAAGGACCAGCTGGACAAC, ACCCAAGTAACCCTTAAAGTCCTGCA). All primers were synthesized by GenScript (Nanjing, China); the mRNA levels of each gene were normalized by GAPDH.

### 2.11 Hematoxylin and Eosin (H&E) staining and immunohistochemistry

Tumor tissues were fixed with 4% (w/v) PFA (Cat# 1004965000, Sigma-Aldrich, Germany) and embedded in paraffin blocks. Sections were cut at 5 μm from paraffin-embedded blocks, deparaffinized in xylene, and rehydrated in graded ethanol. After that, standard H&E staining was performed. For immunohistochemistry, the rehydrated sections were incubated with 3% hydrogen peroxide to block endogenous peroxidase activity. After that, the sections were incubated with rabbit anti-mouse CD31 mAB (Cat# ab182981, Abcam, USA) or rabbit anti-mouse CD8 mAB (Cat# ab217344, Abcam) (1:200 dilution) and HRP-conjugated goat anti-rabbit secondary antibody (Cat# ab205718, Abcam) (1:1000 dilution). Finally, the sections were stained with diaminobenzidine (DAB; Cat# ab64238, Abcam) and counterstained with 37% (w/v) hematoxylin (Cat# ab220365, Abcam).

### 2.12 Statistical analyses

All Statistical analyses were performed using Prism 8 (GraphPad Software Inc., CA, USA). Data are presented as means ± standard deviation (SD). Student's *t*-test or analysis of variance (ANOVA) was used to analyze for differences. Survival curves were plotted according to the Kaplan–Meier method and the statistical significance was calculated using the Log-Rank test. In all statistical analyses, *P* < 0.05 was considered statistically significant.

## 3 Results

### 3.1 Generation and characterization of recombinant oncolytic VVs

**Fig. 1:**
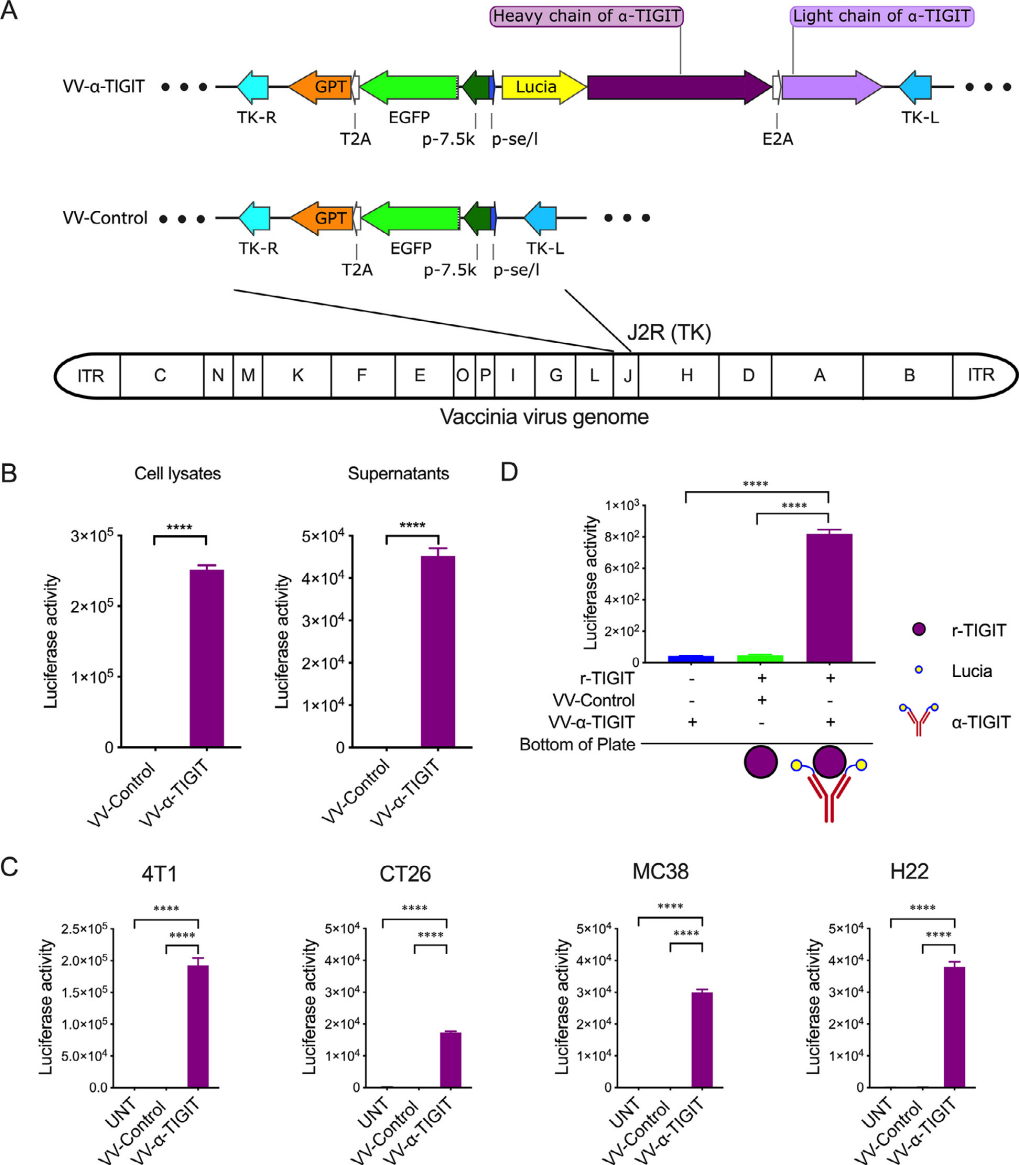
Generation and characterization of recombinant oncolytic VVs. (A) A schematic diagram of the recombinant VVs with (VV-α-TIGIT) or without (VV-Control) α-TIGIT gene. TK-R, right flank sequences of thymidine kinase gene; TK-L, left flank sequences of thymidine kinase gene; GPT, guanine phosphoribosyl transferase; EGFP, enhanced green fluorescent protein; p-7.5k, vaccinia virus p-7.5k early/late promoter; p-se/l, synthesized vaccinia virus early/later promoter; T2A, thoseaasigna virus 2A; E2A, equine rhinitis A virus 2A; (B) Expression and secretion of α-TIGIT in VV-infected Hela-S3 cells. Hela-S3 cells were infected with indicated VVs at MOI of 1 for 24 h, and the levels of α-TIGIT antibody in the cell lysates and supernatants were detected by a luciferase assay. Data are expressed as means ± SD. The experiment was repeated three times. Statistical differences were estimated using the student's *t*-test. ^⁎⁎⁎⁎^*P* < 0.0001. (C) Secretion of α-TIGIT in VV-infected 4T1, CT26, MC38, and H22 cells. The detection of α-TIGIT is similar to that described in (B). Statistical differences were estimated using the ANOVA. ^⁎⁎⁎⁎^*P* < 0.0001. (D) Luciferase-linked immunosorbent assay was used to investigate the binding of the secreted α-TIGIT antibody to the recombinant TIGIT (r-TIGIT) protein. The 96-well plate was coated with r-TIGIT (10 μg/ml), and the supernatants of cells infected with VVs were added to the plate. After three rounds of washing, a luciferase assay was used to verify the binding of the secreted α-TIGIT antibody to the r-TIGIT protein. Data are expressed as means ± SD. The experiment was repeated three times. Statistical differences were estimated using analysis of variance (ANOVA). ^⁎⁎⁎⁎^*P* < 0.0001.

The recombinant VV encoding a fully anti-mouse TIGIT mAB (VV-α-TIGIT) was generated by inserting a p-se/l-derived transcription unit with the antibody-coding sequence into the J2R (TK) locus of the VV genome (Fig. 1**A**). At the same time, an additional p-7.5k-derived transcription unit with a reporter gene (EGFP) and a screening gene (GPT) was also inserted into the J2R locus. The insertion of these foreign genes led to disrupting the TK of the VV. A control VV (named VV-Control) without the α-TIGIT gene insertion was generated analogously (Fig. 1**A**). By using three rounds of GPT screening and two rounds of plaque purification, two purified recombinant clones (VV-α-TIGIT and VV-Control) was selected without parental VV (**Fig. S2A)**, as confirmed by PCR amplification of the target gene and the TK gene (**Fig. S2B)**.

Next, to investigate whether VV-α-TIGIT could infect tumor cells and secret the α-TIGIT, one human cell line Hela-S3 and four murine tumor cell lines (4T1, CT26, MC38, and H22) were infected with VV-α-TIGIT at an MOI of 1. As shown in Fig. 1**B**, the α-TIGIT antibody was efficiently produced and released from VV-α-TIGIT-infected Hela-S3 cells, as detected by luciferase assay. Similarly, the four murine tumor cells infected with VV-α-TIGIT also efficiently secreted the α-TIGIT antibody. Among these cell lines, the 4T1 cell line reached a higher luciferase activity, suggesting a relatively higher level of α-TIGIT secretion (Fig. 1**C)**.

Subsequently, we performed a luciferase-linked immunosorbent assay to explore whether the secreted α-TIGIT could bind to TIGIT. As shown in Fig. 1**D**, the supernatant of Hela-S3 cells infected with VV-α-TIGIT only showed a background level of luciferase activity in the well that was not coated with r-TIGIT. However, in the well pre-coated with r-TIGIT, the supernatant showed a significantly higher luciferase activity (*P* < 0.0001). As expected, the supernatant of Hela-S3 cells infected with VV-Control showed a background level of luciferase activity even in the well pre-coated with r-TIGIT. These results indicate that the secreted α-TIGIT antibody was efficiently bound to TIGIT.

### 3.2 The recombinant oncolytic VVs effectively replicated in tumor cells and lysed the tumor cells

**Fig. 2:**
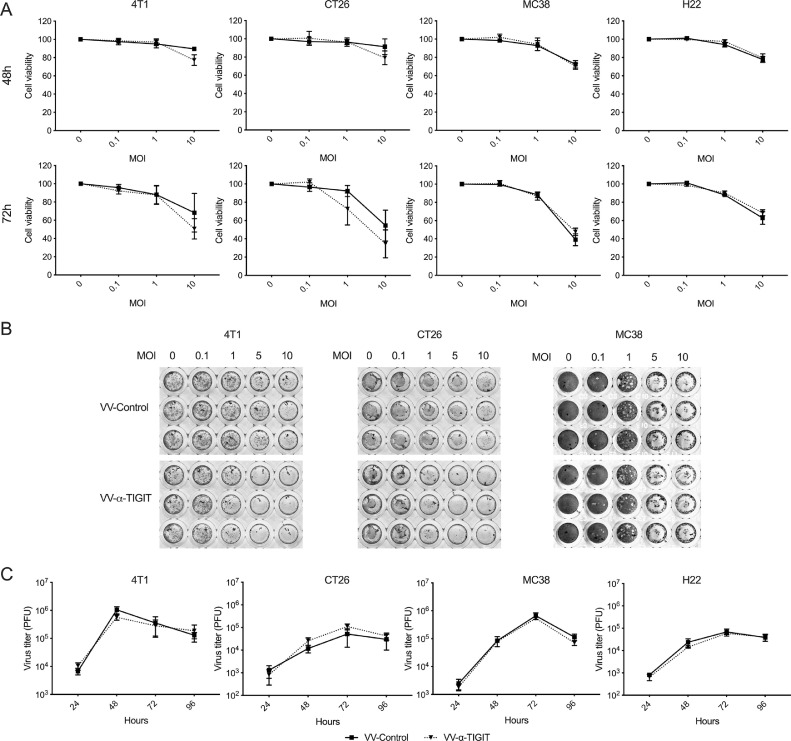
Oncolysis and replication of VVs in tumor cells. **(A)** 4T1, CT26, MC38, and H22 cells were plated into 96-well plates and infected with VVs at the indicated MOI for 48 and 72 h. The cell viability was determined by MTT assay for 4T1, CT26, and MC38 cells and was determined by CCK8 assay for H22 cells. Data represent the mean ± standard deviation (SD) of three independent experiments. **(B)** 4T1, CT26, and MC38 cells were plated into a 96-well plate and infected with VVs at the indicated MOI for 72 h, and oncolytic ability was determined by crystal violet staining. The figure represents one of the three experiments performed. (**C**) 4T1, CT26, MC38, and H22 cells were infected with VVs at MOI of 0.1, cells were harvested at designated time points and progeny viral particles were quantified by titration assays (TCID50).

To evaluate the oncolytic ability of the recombinant VV, 4T1, CT26, MC38, and H22 cells were infected with VVs at different MOIs. The cytotoxicity was assessed by either the MTT or the CCK8 assay after 48 and 72  h of infection (Fig. 2**A**). Both VV-α-TIGIT and VV-Control induced time-dependent and dose (MOI)-dependent killing activity against 4T1, CT26, MC38, and H22 cells. Although VV-α-TIGIT showed a stronger trend of oncolytic activity against 4T1 and CT26 cells at MOI of 10, there was no statistical difference in the oncolytic activity between VV-α-TIGIT and VV-Control (*P* > 0.05). For MC38 and H22 cells, no difference was observed in oncolytic activity between these two VVs. A crystal violet staining assay was also used to confirm the oncolytic ability of the recombinant VV. Consistent with Fig. 2**A,** Both VV-α-TIGIT and VV-Control showed dose (MOI)-dependent oncolytic activity against 4T1, CT26, and MC38 cells (Fig. 2**B**).

To evaluate the replication capacity of the oncolytic VV, 4T1, CT26, MC38, and H22 cells were infected with VV-α-TIGIT and VV-Control at an MOI of 0.1. The replication efficiency was evaluated by a TCID50 method after 24, 48, 72, and 96  h of infection. In all these cells, the two VVs showed time-dependent replication and had similar replication capacity. In 4T1 cells, two VVs reached their highest titers at 48 h after infection, while in the other 3 tumor cells they reached their highest titers at 72 h. (Fig. 2**C**). In summary, compared with VV-Control, the replication and oncolytic ability of VV-α-TIGIT were unimpaired by the transgene.

### 3.3 Influence of VV-infection on the expression of PVR/CD155 in tumor cells

Based on tumor and normal samples from The Cancer Genome Atlas (TCGA) and the Genotype-Tissue Expression (GTEx) databases via GEPIA2 [16], we firstly analyzed the mRNA expression of the main members of TIGIT-PVR/CD155 (**Fig. S3A**) and PD-1/PD-L1 (**Fig. S3B**) signaling in breast cancer (BRCA), colon adenocarcinoma (COAD), rectal carcinoma (READ), and liver hepatocellular carcinoma (LIHC). In COAD and READ, the expression of PVR/CD155, PVRL1/CD111, PVRL2/CD112, TIGIT, and PVRIG/CD112R was significantly higher in tumor tissues than that in normal tissues. In BRCA, the expression of PVRL1/CD111, PVRL2/CD112, TIGIT, and DNAM-1/CD226 in tumor tissues was significantly higher than that in normal tissues, but PVRL3/CD113 showed an opposite expression pattern. In LIHC, the expression of PVR/CD155, PVRL1/CD111, and PVRL2/CD112 was significantly higher in tumor tissues than that in normal tissues. In contrast, the expression of PD-L1 was lower in LIHC tissues than that in normal tissues. In both BRCA and COAD, PD-1 showed a similar expression pattern as TIGIT.

**Fig. 3:**
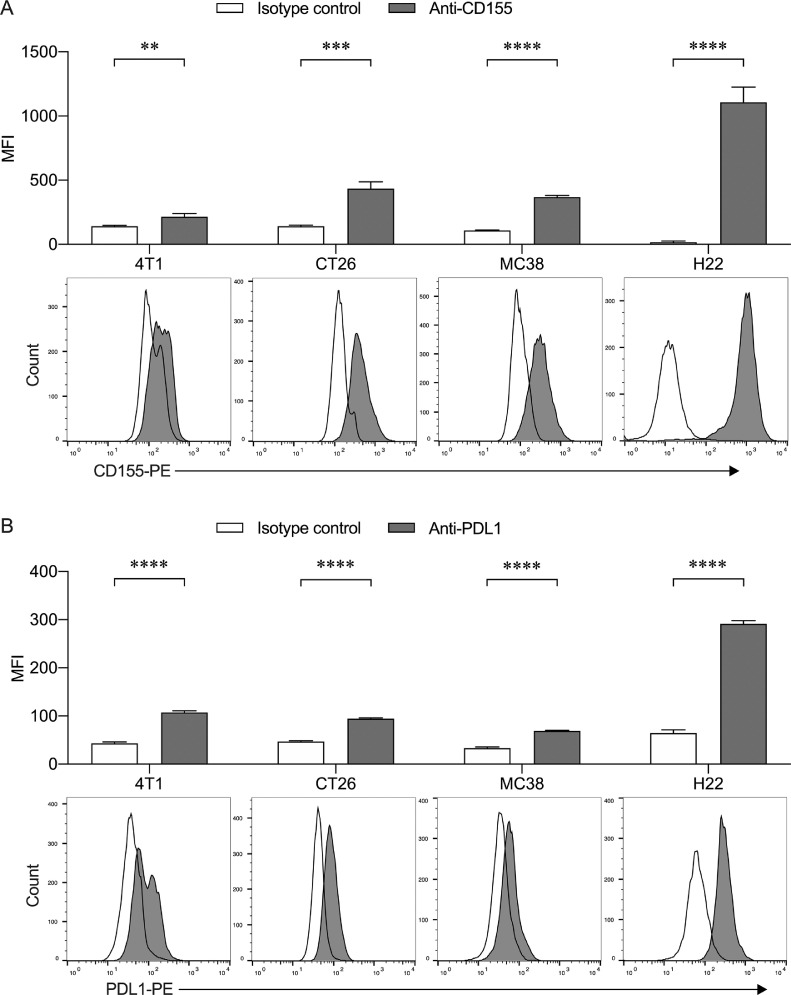
Expression of PVR/CD155 and PD-L1 on tumor cells. Flow cytometry histogram representative of the expression of PVR/CD155 **(A)** and PD-L1 **(B)** on 4T1, CT26, MC38 and H22 cells. To analyze PVR/CD155, PE-conjugated rat-anti-mouse IgG2a was used as an isotype control. To analyze PD-L1, PE-conjugated rat-anti-mouse IgG2b was used as an isotype control. Data are presented as the mean fluorescence intensity (MFI) ± SD of three independent assay. **P < 0.01; ***P < 0.001; ****P < 0.0001.

Subsequently, we investigated the expression of PVR/CD155 and PD-L1 on 4T1, CT26, MC38, and H22 cells by flow cytometry. As shown in Fig. 3**A**, all these tumor cells positively expressed PVR/CD155, in which H22 cells expressed a relatively higher level. Positive expression of PD-L1 was also detected on these cells. However, its mean fluorescence intensity was much lower than PVR/CD155 (Fig. 3**B)**. Finally, we tested whether VV infection affected the expression of PVR/CD155 on these cells. As shown in **Fig. S4**, when tumor cells were infected with VV at MOI of 1 for 48 h, the 4T1 cells showed the highest GFP positive rate (more than 80%), while CT26 and MC38 cells had a moderate GFP positive rate (about 40–50%), and H22 had the lowest GFP positive rate (approximately 20%). Interestingly, the PVR/CD155 expression on the surface of 4T1 cells was down-regulated after the cells were infected with VV, while its expression on the other three tumor cells did not change significantly (**Fig. S4**).

### 3.4 Anti-tumor activity of VV-α-TIGIT on breast cancer models

**Fig. 4:**
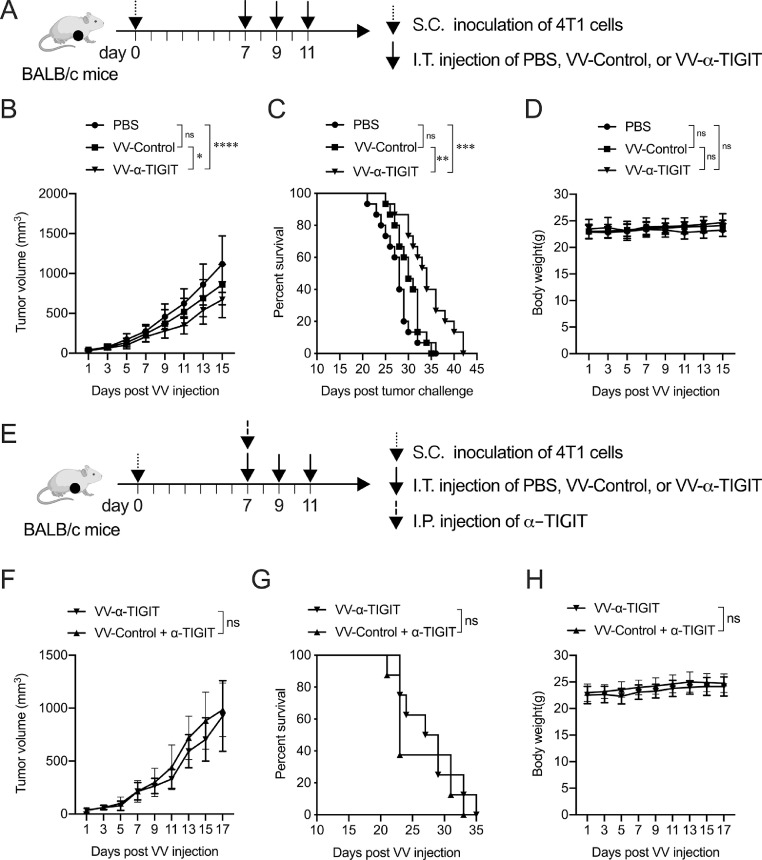
VV-α-TIGIT has enhanced antitumor activity in a breast cancer model. **(A)** The mice treatment scheme. The breast cancer subcutaneous (S.C.) model was established by implantation of 2 × 10^5^ 4T1 cells on the right flank of BALB/c mice. When the tumor volume reached approximately 50 mm^3^ (7 days post tumor inoculation), the tumor-bearing mice were treated with PBS, 1 × 10^7^ PFU of VV-Control, or VV-α-TIGIT at a 2-day interval for 3 times via intratumoral (I.T.) injection. **(B)** Tumor volumes were determined every two days. Data are presented as the mean ± SD of 15 mice for all groups. **(C)** Kaplan–Meier survival curves of tumor-bearing mice treated with PBS, VV-Control, and VV-α-TIGIT. **(D)** The body weight of the mice. Data are presented as the mean ± SD of 15 mice for all groups. **(E)** The mice treatment scheme of monotherapy with VV-α-TIGIT or combination therapy with VV-Control and α-TIGIT. The tumor model was established similar to **(A**). In the combination treatment group, an additional α-TIGIT intraperitoneally (I.P.) treatment was performed during the first intratumoral injection of VV-Control. **(F)** Tumor volume of mice treated with VV-α-TIGIT or VV-Control plus α-TIGIT was determined every two days. Data are presented as the mean ± SD of 8 mice for each group. **(G)** Kaplan–Meier survival curves of tumor-bearing mice treated with VV-α-TIGIT or VV-Control plus α-TIGIT. **(H)** The body weight of the mice. Data are presented as the mean ± SD of 8 mice for each group. Statistical differences in tumor volume and body weight among the groups were evaluated using ANOVA. Statistical differences in survival among the groups were evaluated using the Log-Rank test. ns, no significant differences; *P < 0.05; **P < 0.01; ***P < 0.001; ****P < 0.0001.

To assess the anti-tumor activity of the oncolytic VV *in vivo*, 4T1 tumor-bearing BALB/c mice were treated with PBS, VV-Control, or VV-α-TIGIT every other day for 3 times via intratumoral injection (Fig. 4**A**). Mice treated with VV-α-TIGIT had significantly lower tumor volume compare to mice treated with VV-Control (*P* <0.05) or PBS (*P* <0.0001) (Fig. 4**B**). Mice treated with VV-α-TIGIT survived longer than mice treated with VV-Control (*P* <0.01) or PBS (*P* < 0.001) (Fig. 4**C**). There was no significant difference in tumor volume and survival time between VV-Control- and PBS-treated mice (*P* > 0.05) (Fig. 4**B–C**). No difference was observed in body weight among the three groups of mice (*P* > 0.05) (Fig. 4**D**). These data indicated that VV-α-TIGIT is superior to VV-Control in reducing tumor burden and prolonging survival in the 4T1 breast cancer model.

To compare the therapeutic efficacy of VV-Control plus anti-TIGIT antibody (α-TIGIT) with VV-α-TIGIT monotherapy, 4T1 tumor-bearing mice were treated with VV-Control or VV-α-TIGIT every other day for 3 times via intratumoral injection. In the combination treatment group, additional α-TIGIT was intraperitoneally injected during the first intratumorally injection of VV-Control (Fig. 4**E**). There was no significant difference in tumor volume, survival time, and the body weight of mice between the monotherapy with VV-α-TIGIT and the combination therapy with VV-Control plus α-TIGIT (*P* > 0.05) (Fig. 4**F–H**). These data indicated that monotherapy with VV-α-TIGIT shows a comparable antitumor efficacy to combination therapy with VV-Control plus α-TIGIT.

### 3.5 Anti-tumor activity of VV-α-TIGIT on colon cancer models

**Fig. 5:**
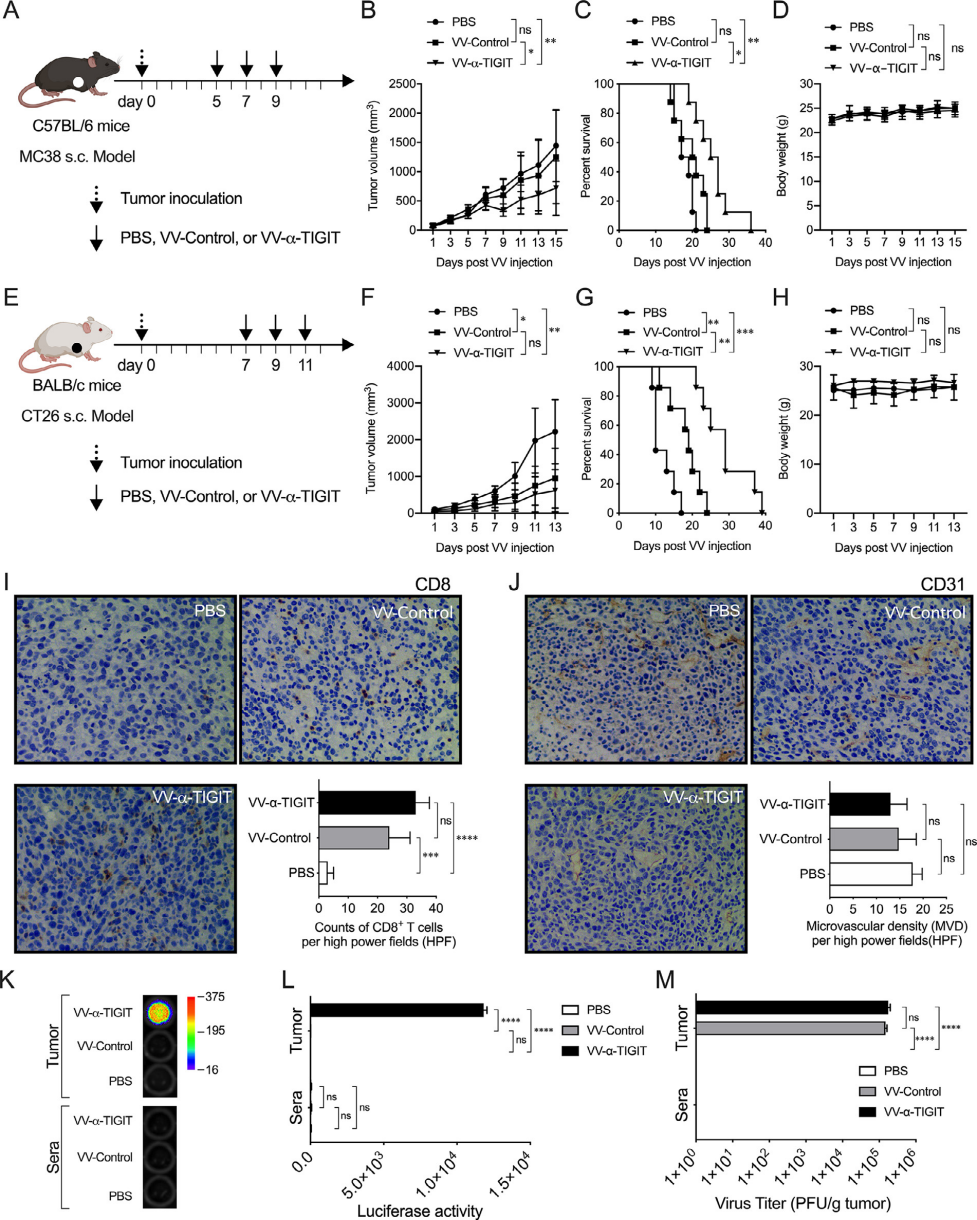
Anti-tumor activity of the recombinant VV on colon cancer models. **(A)** Treatment scheme of MC38 tumor model. The subcutaneous tumor model was established by inoculation of 2 × 10^6^ MC38 cells on the right flank of C57BL/6 mice. Mice were administered intratumorally at the indicated day for 3 times with PBS, 1 × 10^7^ PFU of VV-Control or VV-α-TIGIT. **(B)** Tumor volumes were determined every two days. Data are presented as the mean ± SD of 8 mice for all groups. **(C)** Kaplan-Meier survival curves of tumor-bearing mice treated with PBS, VV-Control, and VV-α-TIGIT. **(D)** The body weight of the mice. Data are presented as the mean ± SD of 8 mice for all groups. **(E)** Treatment scheme of CT26 tumor model. The subcutaneous tumor model was established by inoculation of 5 × 10^5^ CT26 cells on the right flank of BALB/c mice. Mice treatment were similar to (**A**). **(F)** Tumor volumes were determined every two days. Data are presented as the mean ± SD of 8 mice for all groups. **(G)** Kaplan–Meier survival curves of tumor-bearing mice treated with PBS, VV-Control, and VV-α-TIGIT. **(H)** The body weight of the mice. Data are presented as the mean ± SD of 8 mice for all groups. **(I, J)** Immunohistochemistry (IHC) detection of the infiltration of CD8^+^ T cells **(I)** and microvascular density (MVD) **(J)** of the tumor in the CT26 subcutaneous tumor model. The tumor model was established as previously described. When the tumor reached approximately 50 mm^3^, mice were treated i.t. with PBS, 1 × 10^7^ PFU of VV-Control or VV-α-TIGIT. Seven days after VV injection, tumors were collected from mice, and CD8 and CD31 expression were detected by IHC. **(K, L)** The α-TIGIT levels in tumor and blood. Samples of the tumor lysates and sera were prepared as previously described in materials and methods and a luciferase assay was used to detect the levels of secreted α-TIGIT. **(M)** Viral titers in tumor and blood. Samples were collected similar to **(K, L)**, and viral titers were quantified by a TCID50 method. Statistical differences in tumor volume, body weight, CD8^+^ T cells, MVD, luciferase activity, and viral titer among the groups were evaluated using ANOVA. Statistical differences in survival among the groups were evaluated using the Log-Rank test. ns, no significant differences; *P < 0.05; **P < 0.01; ***P < 0.001.

To assess the anti-tumor activity of the oncolytic VV on MC38 colon cancers, the tumor-bearing C57BL/6 mice were treated with PBS, VV-Control, or VV-α-TIGIT at 2-day intervals for 3 times via intratumoral injection (Fig. 5**A**). Similar to the 4T1 model, the tumor volume of mice treated with VV-α-TIGIT was significantly lower than that of mice treated with VV-Control or PBS (*P* < 0.05; *P* < 0.01) (Fig. 5**B**). Mice treated with VV-α-TIGIT survived longer than mice treated with VV-Control or PBS (*P* <0.05; *P* < 0.01) (Fig. 5**C**). There was no difference in tumor volume and survival time between the VV-Control- and PBS-treated mice (*P* > 0.05) (Fig. 5**A, B**).

We also investigated the anti-tumor activity of VV-α-TIGIT in a CT26 colon cancer model. The mice treatment scheme was similar to Fig. 4A (Fig. 5**E**). Compared with PBS, the administration of both VV-Control and VV-α-TIGIT significantly inhibited tumor growth (*P* < 0.05; *P* < 0.01) and prolonged the survival of mice (*P* < 0.01; *P* < 0.001) (Fig. 5**F, G**). Although the mice treated with VV-α-TIGIT only showed a trend of lower tumor volume compare to mice treated with VV-Control (*P* > 0.05) (Fig. 5**F**), these mice demonstrated a significantly longer survival time than mice treated with VV-Control (*P* <0.01) (Fig. 5**G**). In these two models, no significant difference was observed in body weight among the three groups (*P* > 0.05) (Fig. 5**D, H**). In addition, no damage to tissues including heart, liver, spleen, lung, kidney, small intestine, muscle and brain was observed in all the examined mice (**Fig. S6**).

Consistent with the above results, CD8^+^ T cells were highly infiltrated in the CT26 tumors treated with VV-Control or VV-α-TIGIT than those treated with PBS (*P* < 0.001; *P* < 0.0001) (Fig. 5**I**). Mice treated with VV-α-TIGIT showed a trend of higher infiltration of CD8^+^ T cells compare to mice treated with VV-Control (*P* > 0.05) **(**Fig. 5**I**). Although tumor microvascular density (MVD) showed a decreasing trend in the group of mice treated with VV-α-TIGIT compare to VV-Control or PBS, no significant difference was observed between these three groups **(**Fig. 5**J**). Compare to PBS and VV-Control, high levels of α-TIGIT were released in tumors of mice treated with VV-α-TIGIT as detected by luciferase activity (*P* < 0.0001; *P* < 0.0001) (Fig. 5**K, L**). However, the α-TIGIT was not detected in the sera of all the three groups of mice, suggesting that it was mainly secreted locally in the tumor (Fig. 5**K, L**). As expected, comparable virus titers were detected in tumor tissues of mice treated with VV-α-TIGIT or VV-Control. In contrast, no virus was detected in the sera of the three groups of mice (Fig. 5**M**). Taken together, these data indicate that VV-α-TIGIT has a better anti-tumor effect than VV-Control in colon cancer models.

### 3.6 Anti-tumor activity of VV-α-TIGIT on hepatocellular carcinoma model

**Fig. 6:**
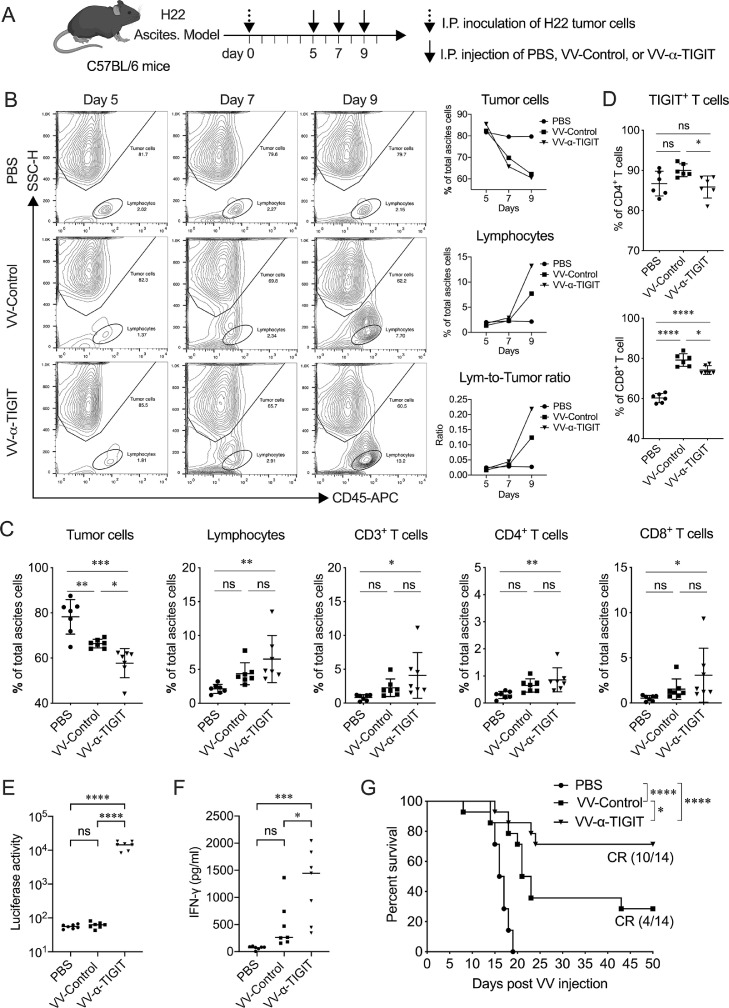
VV-α-TIGIT enhanced immune-cell infiltration and activation in the tumor microenvironment. **(A)** The treatment scheme. The tumor model was established by intraperitoneally (I.P.) inoculation of 5×10^6^ H22 cells in C57BL/6 mice. After ascites were formed, the tumor-bearing mice were treated with PBS, 1 × 10^7^ PFU of VV-Control or VV-α-TIGIT at a 2-day interval 3 times via I.P. injection. **(B)** Dynamic detection of the proportion of the tumor cells and lymphocytes in the ascites by flow cytometry. **(C, D)** Flow cytometric analysis of the proportion of the tumor cells, lymphocytes, and their subsets in the ascites at day 4 after the first VV-α-TIGIT treatment. **(E)** Kaplan–Meier survival curves of tumor-bearing mice treated with PBS, VV-Control, or VV-α-TIGIT (*n*=14). **(F)** The α-TIGIT levels in ascites. Ascites were harvested on day 4 after the first VV-α-TIGIT treatment and a luciferase assay was used to detect the levels of secreted α-TIGIT in ascites. **(G)** The concentration of murine IFN-γ was detected by an ELISA assay in the ascites of mice on day 4 after the first VV-α-TIGIT treatment. Statistical differences in cell proportion, luciferase activity, and IFN-γ concentration in the ascites were evaluated using ANOVA. Statistical differences in survival of mice among the groups were evaluated using the Log-Rank test. ns, no significant differences; *P < 0.05; **P < 0.01; ***P < 0.001; ****P < 0.0001.

To evaluate the effect of oncolytic VV on hepatocellular carcinoma, a model of H22 ascites tumor was established in C57BL/6 mice. When ascites formed, mice were treated with PBS, VV-Control, or VV-α-TIGIT at a 2-day interval 3 times via intraperitoneal injection (Fig. 6**A**). Flow cytometry results showed that the tumor cells in the ascites of mice treated with VV-Control or VV-α-TIGIT gradually decreased compared to mice treated with PBS. However, lymphocytes in the ascites of mice treated with VV-Control or VV-α-TIGIT gradually increased compared to mice treated with PBS. The change of lymphocyte-to-tumor-cell ratio showed the same trend as lymphocytes **(**Fig. 6**B)**. On the 9th day after the establishment of the tumor model (2 days after the second VV treatment, that is, before the third VV treatment), the proportion of tumor cells in the ascites of mice treated with VV-Control or VV-α-TIGIT was significantly lower than that of mice treated with PBS (*P* <0.01; *P* < 0.001). Of note, VV-α-TIGIT-treated mice showed a significantly lower proportion of tumor cells in the ascites compare to VV-Control-treated mice (*P* <0.05) (Fig. 6**C**). Compared with PBS-treated mice, VV-α-TIGIT-treated mice had a significantly higher proportion of lymphocytes, CD3^+^, CD4^+^, and CD8^+^ T cells in ascites (*P* <0.01; *P* <0.05; *P* <0.01; *P* <0.05) (Fig. 6**C**). Compared with mice treated with VV-Control, mice treated with VV-α-TIGIT showed a trend of higher infiltration of lymphocytes, CD3^+^, CD4^+^, and CD8^+^ T cells, although there was no significant diffidence (*P* > 0.05) (Fig. 6**C**). Compared with PBS, mice treated with VV-Control only showed a tendency to increase the proportion of TIGIT-positive CD4^+^ T cells (*P* > 0.05), whereas dramatically increased the proportion of TIGIT-positive CD8^+^ T cells (*P* < 0.0001) (Fig. 6**D**). However, VV-α-TIGIT treatment significantly reversed the increase of TIGIT-positive CD4^+^ and CD8^+^ T cells, making them lower than the VV-Control treatment group (*P* < 0.05, *P* < 0.05) (Fig. 6**D**). At the same time, mice treated with VV-α-TIGIT showed higher luciferase activity in ascites than mice treated with PBS or VV-Control (*P* < 0.0001; *P* < 0.0001), indicating that VV-α-TIGIT can express higher levels of α-TIGIT in vivo (Fig. 6**E**). Furthermore, mice treated with VV-α-TIGIT showed a higher level of IFN-γ in ascites than mice treated with PBS or VV-Control (*P* < 0.001; *P* < 0.05) (Fig. 6**F**). Consist with these results, mice treated with VV-Control or VV-α-TIGIT survived longer than mice treated with PBS (*P* < 0.0001), and mice treated with VV-α-TIGIT survived longer than mice treated with VV-Control (*P* <0.05) (Fig. 6**G**). Of note, mice treated with VV-α-TIGIT exhibited approximately 70% (10/14) of tumor complete regression (CR), whereas mice treated with VV-Control only showed approximately 30% (4/14) of tumor CR, and mice treated with PBS was not observed with tumor CR (Fig. 6**G**). These data indicate that VV-α-TIGIT has a better anti-tumor effect than VV-Control in the hepatocellular carcinoma model.

We also evaluate the effect of VV-α-TIGIT on immune-cell infiltration and activation in another H22 ascites tumor model in BALB/c mice (**Fig. S5**). The mice treatment scheme was similar to Fig. 6**A**. On day 2 post the first VVs injection, there was no significant difference in terms of lymphocytes, NK cells, CD4^+^ T cells, CD8^+^ T cells among the three groups of mice (*P* > 0.05) (**Fig. S5A**). However, on day 2 post the third VV injection, VV-α-TIGIT significantly increased the proportion of lymphocytes in ascites compared to VV-Control and PBS (*P* < 0.05; *P* < 0.0001) (**Fig. S5A, B**). This increase is mainly contributed by the increase of the CD4^+^ and CD8^+^ T cells (*P* < 0.01 and *P* < 0.001 vs PBS), not by the NK cells (all *P* > 0.05 vs VV-Control or PBS) (**Fig. S5A**). Compared to PBS and VV-Control, the mRNA expression of INF-γ, IL-2, and IL-6 in ascites cells of mice treated with VV-α-TIGIT showed increasing trends, while the mRNA expression of IL-10 showed a decreasing trend (**Fig. S5C**). Taken together, these data indicate that VV-α-TIGIT has the ability to recruit and activate T cells in the tumor microenvironment and reduce the TIGIT positive immune suppressive T cells.

### 3.7 The in vivo anti-tumor activity of VV-α-TIGIT depends on CD8^+^ T cells

**Fig. 7:**
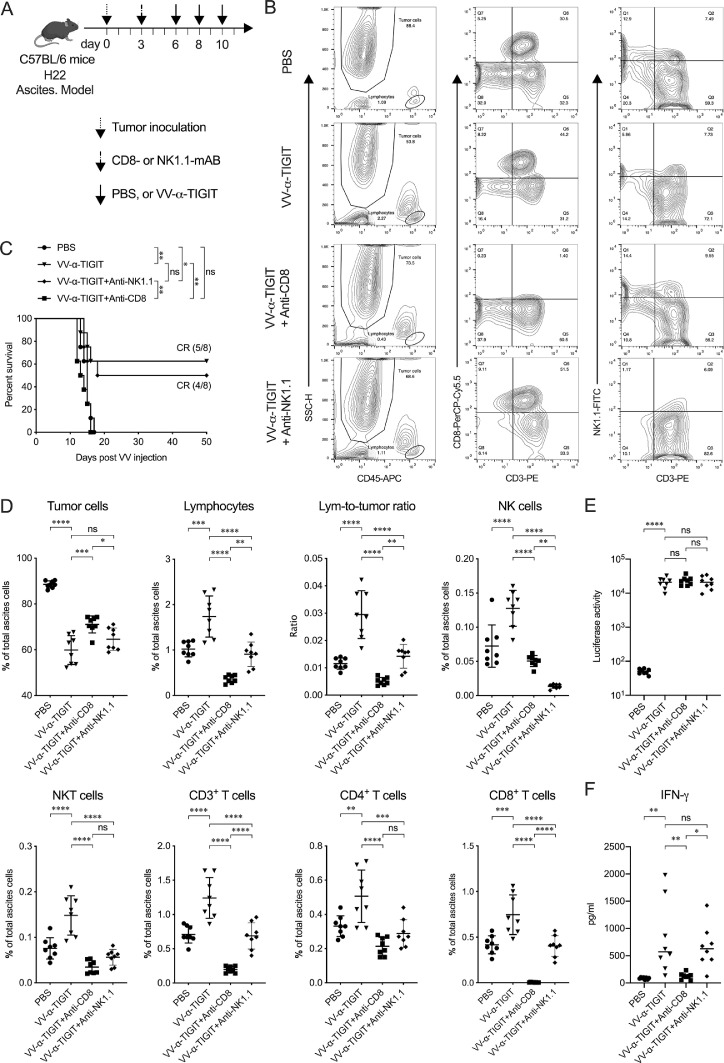
The in vivo anti-tumor activity of VV-α-TIGIT depends on CD8^+^ T cells. **(A)** Schematic diagram of the experiment for CD8^+^ T or NK cell depletion. Ascites tumor model was established as previously described. Three days post tumor inoculation, mice were injected intraperitoneally (I.P.) with 500 ug of anti-mouse CD8α or anti-mouse NK1.1 mAB per mouse. **(B)** The depletion of CD8^+^ T and NK cells was confirmed by detecting the corresponding cells in ascites by flow cytometry 5 days post the mAB injection. **(C)** Kaplan–Meier survival curves of tumor-bearing mice treated with PBS, VV-α-TIGIT, VV-α-TIGIT plus anti-CD8α, or VV-α-TIGIT plus anti-NK1.1. **(D)** Flow cytometric analysis of the proportion of the tumor cells, lymphocytes, and their subsets in the ascites. **(E)** The level of α-TIGIT in ascites was detected by a luciferase assay on day 4 after the first VV-α-TIGIT treatment. **(F)** The concentration of murine IFN-γ was detected by an ELISA assay in the ascites of mice on day 4 after the first VV-α-TIGIT treatment. Statistical differences in cell proportion, luciferase activity, and IFN-γ concentration in the ascites were evaluated using ANOVA. Statistical differences in survival of mice among the groups were evaluated using the Log-Rank test. ns, no significant differences; *P < 0.05; **P < 0.01; ***P < 0.001; ****P < 0.0001.

CD8^+^ T and NK cells are two common immune cells associated with the therapeutic effect of oncolytic VV. To address the involvement of these two types of cells in mediating the anti-tumor activity of VV-α-TIGIT, we depleted either CD8^+^ T or NK cells of mice and analyzed the impact of the depletion of these two types of cells on the therapeutic benefit by monitoring survival in H22 ascites tumor model (Fig. 7**A**). The depletion of CD8^+^ T and NK cells was confirmed by detecting the corresponding cells in ascites by flow cytometry 5 days post the antibody injection (Fig. 7**B**). As shown in Fig. 7**C**, the antitumor effect of VV-α-TIGIT was completely abrogated by the depletion of CD8^+^ T cells, whereas the depletion of NK cells had no obvious impact. Consistent with these results, the proportion of tumor cells in the ascites of CD8-depleted mice was significantly higher than that of non-depleted or NK-depleted mice after treated with VV-α-TIGIT (*P* <0.001; *P* <0.05) (Fig. 7**D**). As expected, the proportion of CD8^+^, CD3^+^, NKT, and lymphocytes was significantly decreased in ascites of CD8-depleted mice and the proportion of NK, NKT, and lymphocytes was significantly decreased in ascites of NK-depleted mice compared to non-depletion mice. Interestingly, CD8-depletion could lead to a significant decrease of NK, NKT, and CD4^+^ T cells and NK-depletion also led to a decrease of CD3^+^, CD4^+^, and CD8^+^ T cells. Consistent with the previous results, mice treated with VV-α-TIGIT showed higher luciferase activity (represent α-TIGIT level) in ascites than mice treated with PBS (*P* < 0.0001) (Fig. 7**E**). Depleted either CD8^+^ T or NK cells of mice had no impact on the secretion α-TIGIT as detected by luciferase activity (Fig. 7**E**). However, the concentration of IFN-γ was significantly decreased in the ascites of CD8-depleted mice (Fig. 7**F**), suggesting that CD8+T cells were a primary source of IFN-γ. These results indicated that CD8^+^ T cell was a critical immune cell type that could affect the tumor microenvironment of VV treatment and the in vivo anti-tumor activity of VV-α-TIGIT was CD8^+^ T cells dependent.

### 3.8 Treatment of mice with VV-α-TIGIT established long-term tumor-specific immunological memory

**Fig. 8:**
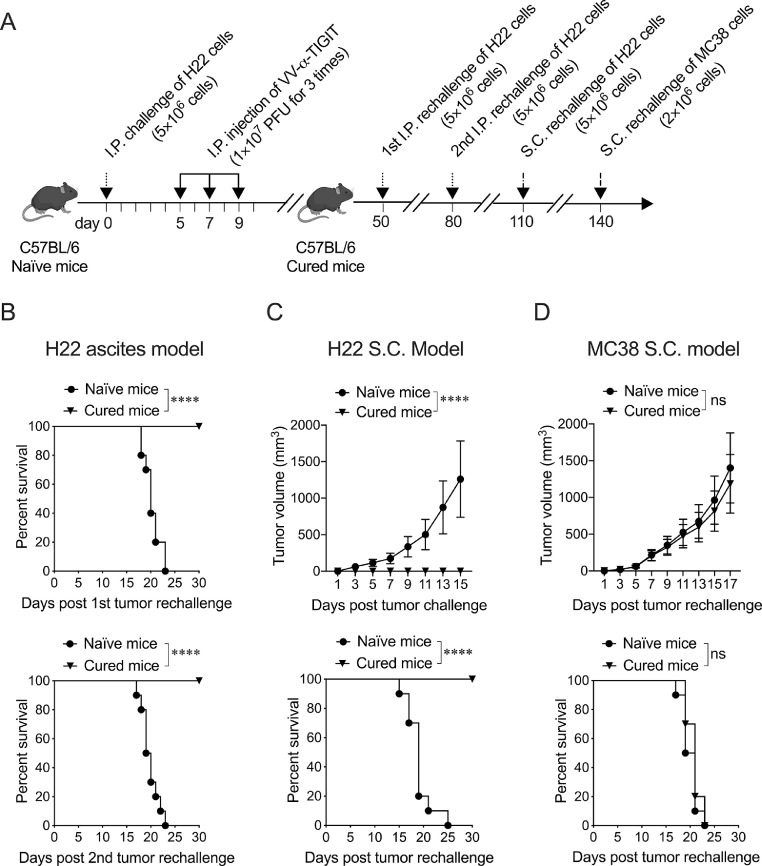
Treatment of mice with VV-α-TIGIT established long-term tumor-specific immunological memory. **(A)** Schematic diagram of tumor rechallenge. Ascites tumor model was established and treated as previously described. Mice cured of H22 by VV-α-TIGIT were twice intraperitoneally and once subcutaneously inoculated with the H22 cells, and once subcutaneously inoculated with MC38 cells at the indicated time. **(B)** Kaplan-Meier survival curves of tumor-naïve or cured mice twice intraperitoneally rechallenged with H22 cells (*n*=10). **(C)** Tumor volumes and Kaplan–Meier survival curves of tumor-naïve or cured mice subcutaneously rechallenged with H22 cells (*n*=10). **(D)** Tumor volumes and Kaplan–Meier survival curves of tumor-naïve or cured mice subcutaneously rechallenged with MC38 cells (*n*=10). Statistical differences in tumor volume were evaluated using ANOVA. Statistical differences in survival were evaluated using the Log-Rank test. ns, no significant differences; ****P < 0.0001.

To investigate the long-term persistence of the immunological antitumor memory, mice cured of H22 by VV-α-TIGIT were twice intraperitoneally and once subcutaneously inoculated with the same tumor cells, and once subcutaneously inoculated with MC38 cells. The mice treatment scheme was showed in Fig. 8**A**. After the first and second intraperitoneal inoculation with H22, all the age-matched tumor-naïve mice developed cancerous ascites and died, while cured mice had no ascites formation and death (Fig. 8**B**). After H22 cells were inoculated subcutaneously, all age-matched tumor-naïve mice developed subcutaneous tumors, whereas mice previously cured of intraperitoneal H22 tumors by VV-α-TIGIT rejected subcutaneously inoculated H22 tumors. (Fig. 8**C**). After MC38 cells were inoculated subcutaneously, both mice cured of H22 by VV-α-TIGIT and age-matched tumor-naïve mice developed subcutaneous tumors (Fig. 8**D**). No differences in tumor volume and survival were observed between these two groups of mice (*P* > 0.05) (Fig. 8**D**). These data indicated that the treatment of tumor-bearing mice with VV-α-TIGIT established long-term tumor-specific immunological memory.

## 4 Discussion

Patients with immune “cold” cancer hardly respond to immunotherapy such as immune checkpoint blockades, because these therapies rely on the infiltration of functional immune cells in the tumor [17]. Oncolytic virotherapy using either a natural virus or a genetically engineered virus provides an approach to convert a “cold” or poorly immunotherapy responsive tumor into a “hot” or immunotherapy responsive tumor [18]. However, its therapeutic potential is affected by the presence of multiple immunosuppressive components in the TME [19]. In the present study, we generated a novel oncolytic VV, VV-α-TIGIT, in which the VV is engineered to encode an anti-TIGIT mAB to overcome this obstacle. This oncolytic VV efficiently infected the tumor cells, replicated, lysed tumor cells, and secreted functional anti-TIGIT mAB. Intratumorally injection of VV-α-TIGIT enhanced anti-tumor efficacy in several subcutaneous tumor models in mice compared to VV-Control. Furthermore, intraperitoneal injection of VV-α-TIGIT achieved complete tumor regression in the ascites tumor model. Additionally, the mice treated with VV-α-TIGIT resulted in the recruitment and activation of T cells in TME. Our results confirm the concept that armed VV with immune checkpoint blockade enhanced the activation of immune responses triggered by virotherapy and the blocking of immunosuppressive responses is essential for the therapeutic benefit [20].

VV is a large DNA virus with a genome size of approximately 190 kb that is able to insert long fragments of foreign genes [21]. Therefore, in the present study, a full-length anti-TIGIT mAB gene fragment could be easily inserted into the genome of VV. However, due to its large genome size, the only way to obtain a recombinant VV with a foreign gene is the homologous recombination of a shuttle plasmid carrying the foreign gene with the parental virus [22]. This makes the subsequent steps to purify the virus (removing the parental virus) complicated. In this study, we used a combination of GPT resistance screening and fluorescent plaque purification to establish a fast and efficient method for purifying recombinant viruses (**Fig. S1**). The method of this article provides an important value for the establishment of similar recombinant VVs.

We showed that the tumor cells infected with the oncolytic VV successfully expressed and secreted antibodies, which effectively bind to TIGIT *in vitro*. Previously studies have reported that oncolytic viruses (such as adenovirus, or VVs) have been successfully designed for the expression of full-length mAB [13, 23]. In these studies, the expression of the heavy and light chain is driven by two separate promoters [13] or by a single promoter with the insertion of an internal ribosome entry site (IRES) between the two chains [23]. This makes it hard to maintain the equimolarity of the heavy and light chain, which may affect the efficiency of antibody assembly. In the present study, we used a 2A peptide to link the heavy and light chain, which ensured the equimolarity of the two chains of the antibody, providing another tandem way of using a single promoter to produce full-length antibodies. Moreover, we labeled the luciferase reporter on the α-TIGIT antibody, providing a simple method for quantifying antibodies by detecting the luciferase activity.

It has been proved that the effectiveness of immunotherapy with ICI is related to the immune status of the tumor microenvironment, and tumors lacking lymphocyte infiltration have a weaker response to ICI [24]. Therefore, the ability of OVs to transform tumors into an environment with higher immune cell content should lead to a better therapeutic response to ICI. The combined use of local injection of OVs and systemic administration of anti-PD-1 mAB has demonstrated increased efficacy compared with either monotherapy [5, 25]. Alternatively, engineered OVs expressing anti-PD-1/PD-L1 mAB were developed and showed equal anti-tumor effects as the combined use of OVs and antibodies [9, 13]. Similar to targeting PD-1, we found that monotherapy with engineered OVs encoding α-TIGIT showed a comparable therapeutic effect as the combined treatment of α-TIGIT and OVs in the breast cancer model. However, the antibody we use to modify VV is a hamster-mouse chimeric antibody, and the antibody administered systemically is rat anti-mouse monoclonal antibodies, our results are difficult to prove which combination is better. Future study needs to use the same antibody as the systemic injection to genetically modify VV to overcome the shortcomings of our research. Nevertheless, since the combination of OVs and ICIs might increase medical costs to health-care systems [15], the engineered VV that encodes ICI seems to provide an alternative combination strategy.

In addition to direct oncolysis, the recruitment and activation of immune cells, especially CD8^+^ T cells, is another major anti-tumor mechanism of oncolytic viruses [26, 27]. In our study, the CT26 subcutaneous tumor was confirmed as a “cold” tumor with little CD8^+^ T cell infiltration, which may partly explain the limited anti-tumor efficacy of the monotherapy with TIGIT blockade in the previous studies [28, 29]. In the mouse H22 ascites tumor model, the proportion of lymphocytes in the ascites of mice without oncolytic VV treatment is only less than 1%, which also confirmed a “cold” immune microenvironment. In both subcutaneous and ascites tumor models, the CD8^+^ T cell infiltration of mice treated with VV-α-TIGIT was much higher than that of mice treated with PBS, indicating that VV-α-TIGIT has a strong function of recruiting T cells, making the “cold” tumors “hot”. Although the VV-α-TIGIT treated mice only showed a trend of more CD8+ T cell infiltration than VV-control, these mice showed significantly higher levels of IFN-γ and a lower proportion of TIGIT-positive CD8+ T cells in the ascites, indicating a higher degree of CD8^+^ T cell activation. It is possible that the activation of CD8^+^ T cells further enhances the secretion of inflammatory cytokines and chemokines, so that immune cells are further recruited into the tumor, resulting in anti-tumor efficacy superior to VV-Control. The depletion of CD8^+^ T cells eliminated the therapeutic efficacy of VV-α-TIGIT, which was accomplished by the reduction of lymphocyte subsets (T, NK, NKT) and IFN-γ secretion, indicating that the activation of CD8^+^ T cells has an indispensable role in anti-cancer immunity induced by VV-α-TIGIT. Previous studies have shown that TIGIT is a checkpoint for NK cells, and blocking TIGIT can prevent NK cell exhaustion and trigger effective anti-tumor immunity [30]. In our study, the depletion of NK cells did not eliminate the therapeutic efficacy of VV-α-TIGIT, suggesting NK cells did not participate in the therapeutic effect of VV-α-TIGIT.

Previous studies have demonstrated that engagement of TIGIT with CD155 can deliver an inhibitory signal, leading to increased secretion of IL-10 in tumor cells [31] and dendritic cells (DCs) [32]. In contrast, the engagement of TIGIT by its ligands resulted in decreased IL-2 production [33]. In the present study, VV-α-TIGIT treatment can reduce IL-10 and increase IL-2 mRNA expression in the ascites cells, which is consistent with the increased α-TIGIT that has the function of blocking TIGIT/CD155 interaction.

We found that VV-α-TIGIT can completely clear the tumor cells and produce long-term immune memory in the ascites tumor model, whereas it could not completely clear the tumor in subcutaneous tumor models. We speculate that the possible reasons are as follows: (1) Although H22 cells derived from C3H/HeJ mice can form tumors in C57BL/6 mice and never undergo regression, they are highly immunogenic in C57BL/6 mice; (2) Compared with solid tumors, the liquid environment of ascites tumors may be more conducive to the diffusion of cytokines and the migration of activated T cells, which causes these T cells to contact and kill tumor cells more effectively; (3) Compared with ascites tumors, subcutaneous tumors may have a higher heterogeneous immune microenvironment, and VV treatment may lead to overexpression and activation of other immune checkpoints, such as PD-1/PD-L1 signaling; (4) compare to 4T1, CT26 or MC38 cells, H22 cells expression relative higher level of PVR, which making these model more sensitive to VV-α-TIGIT therapy. Moreover, we also found that PD-L1 was positively expressed on these cells, although at a relatively lower level compared to PVR, which might reduce the therapeutic efficacy. A recent study has shown that intratumoral delivery of an oncolytic HSV encoding an scFv against PD-1 synergizes with TIGIT blockade [15]. Further combinatorial strategies should be designed and investigated to augment the anti-tumor efficacy of VV-α-TIGIT.

In conclusion, we show for the first time that the VV engineered with α-TIGIT is an effective strategy for oncolytic immunotherapy by combining viral oncolysis and intratumorally expression of immune checkpoint antibodies. This novel strategy can be extended to other immune checkpoint antibodies and can provide information on the optimal design of novel antibody-armed oncolytic viruses for cancer immunotherapy.

## Funding

Jiwu Wei: National Natural Science Foundation of China (81773255 and 81472820); Jie Dong: National Natural Science Foundation of China (81700037); Science and Technology Innovation Foundation of Nanjing University (14913414); Natural Science Foundation of Jiangsu Province of China (BK20171098).

## Declaration of Interests

The authors disclose no conflicts of interest.

## Appendix Supplementary materials

Image, application 1
