# Supplementary material for: Enhanced antitumor efficacy of a novel oncolytic vaccinia virus encoding a fully monoclonal antibody against T-cell immunoglobulin and ITIM domain (TIGIT)
Source: eBioMedicine. 2021 Feb 10;64:103240. doi: 10.1016/j.ebiom.2021.103240 (PMC7878184; doi:10.1016/j.ebiom.2021.103240)
Supplement: Supplementary file 1 [file mmc1.pdf]

Supplementary figures

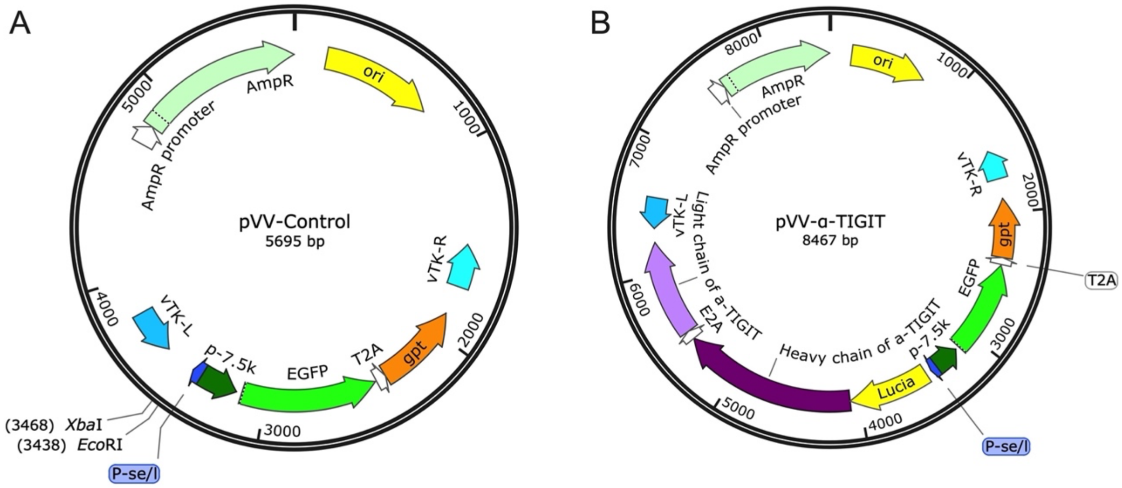

Fig. S1 Map of the shuttle plasmid for construction of the recombinant VVs.

A. Plasmid map of pVV-Control; B. Plasmid map of pVV-α-TIGIT.

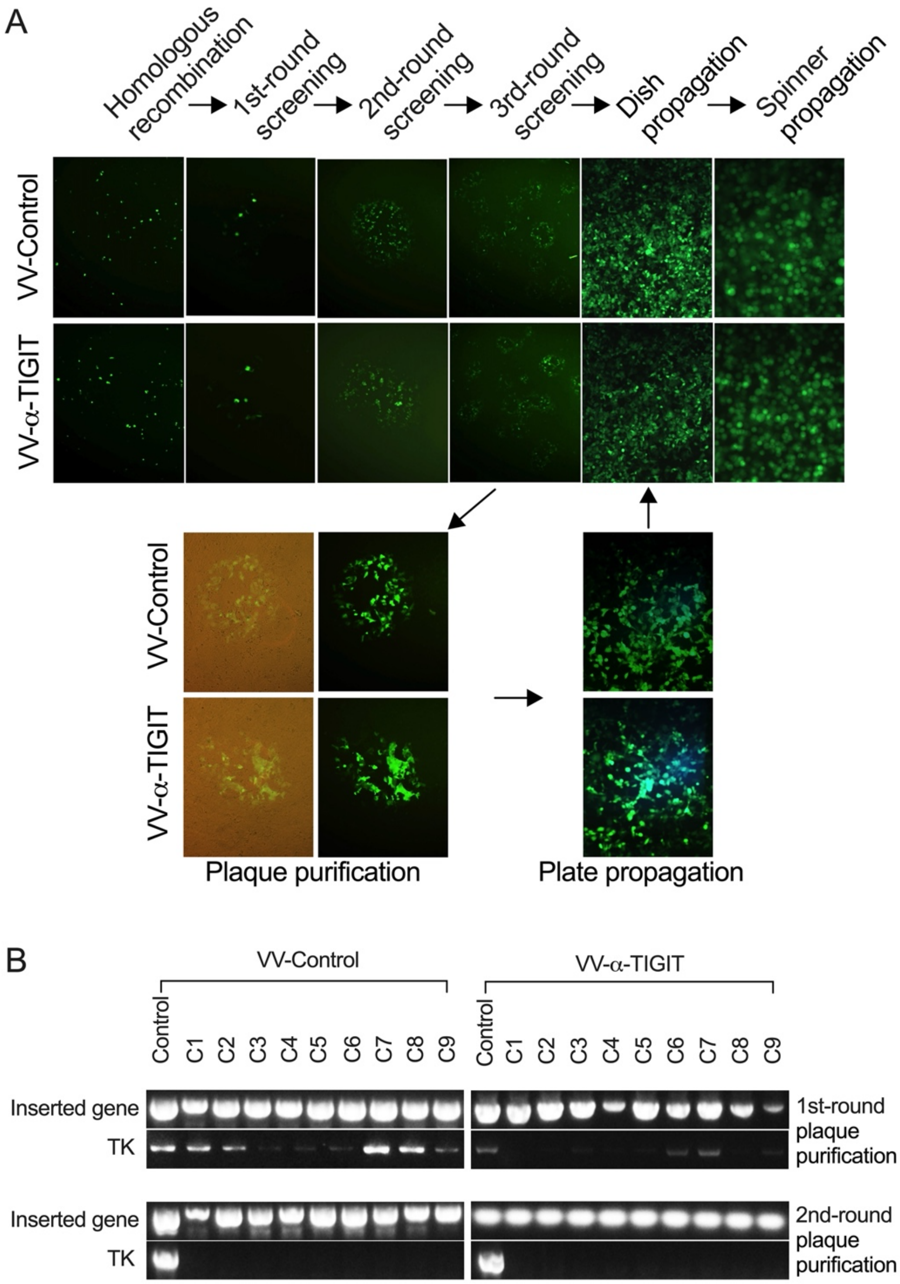

Fig. S2 Generation of recombinant oncolytic VV. A. Flow chart of the recombinant VV construction, purification, and expansion. B. The inserted gene and the viral TK gene were amplified to identify whether the recombinant virus was adulterated with the parental VV (WR). In the first rounds of plaque purification, most of the clones contain parental VV. However, in the second round of plaque purification, none of the clones contain parental VV.

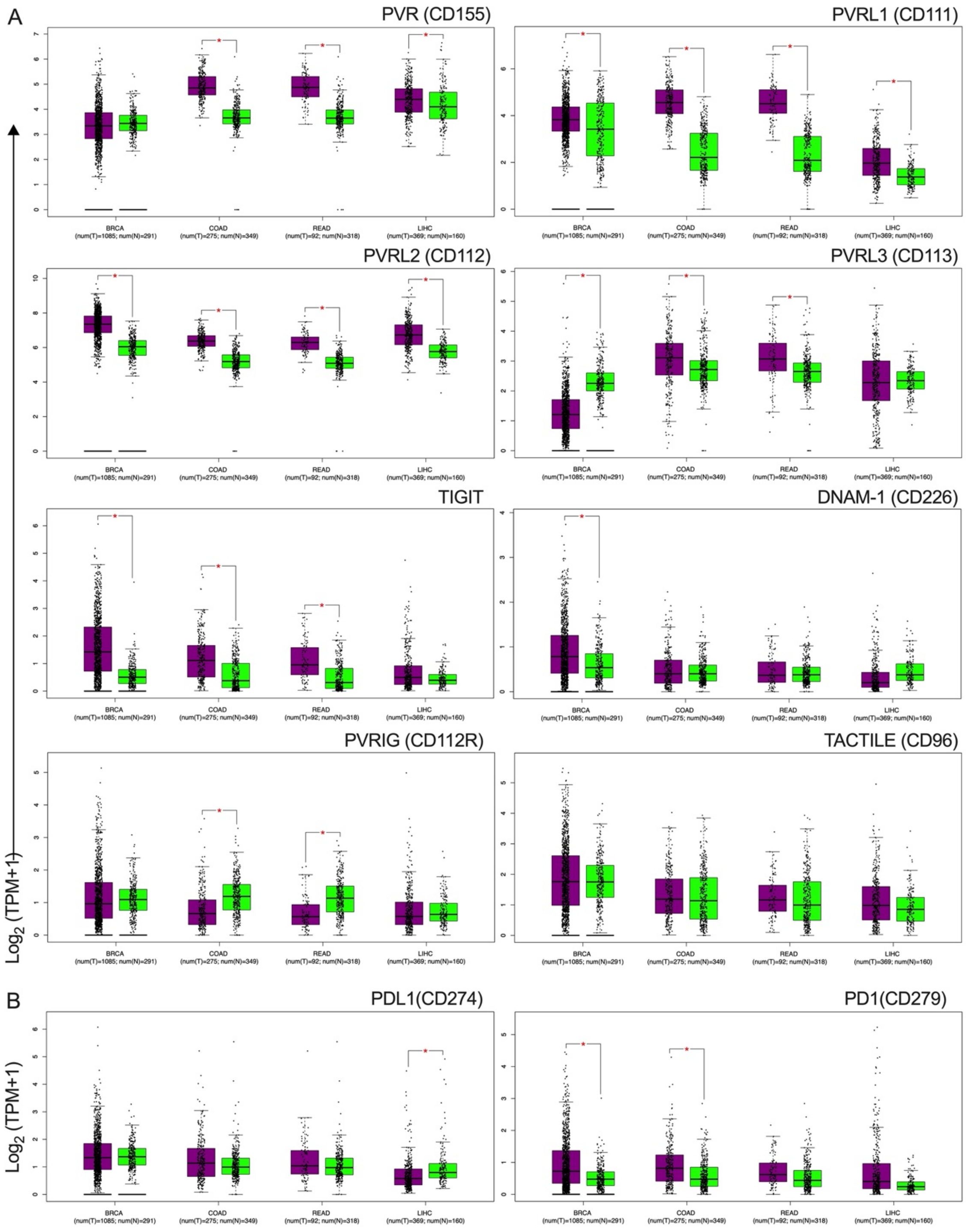

Fig. S3 The mRNA expression level of the main members of TIGIT-PVR/CD155 and PD1-PDL1 signaling. A. The expression level of the main members of TIGIT-PVR/CD155 signaling. B. The expression level of PD1 and PDL1. Data are analyzed in tumor and normal samples from the TCGA and the GTEx databases via GEPIA2 (http://gepia2.cancer-pku.cn). BRCA, breast cancer; COAD, colon adenocarcinoma; READ, rectal carcinoma; LIHC, liver hepatocellular carcinoma. **P*<0.05.

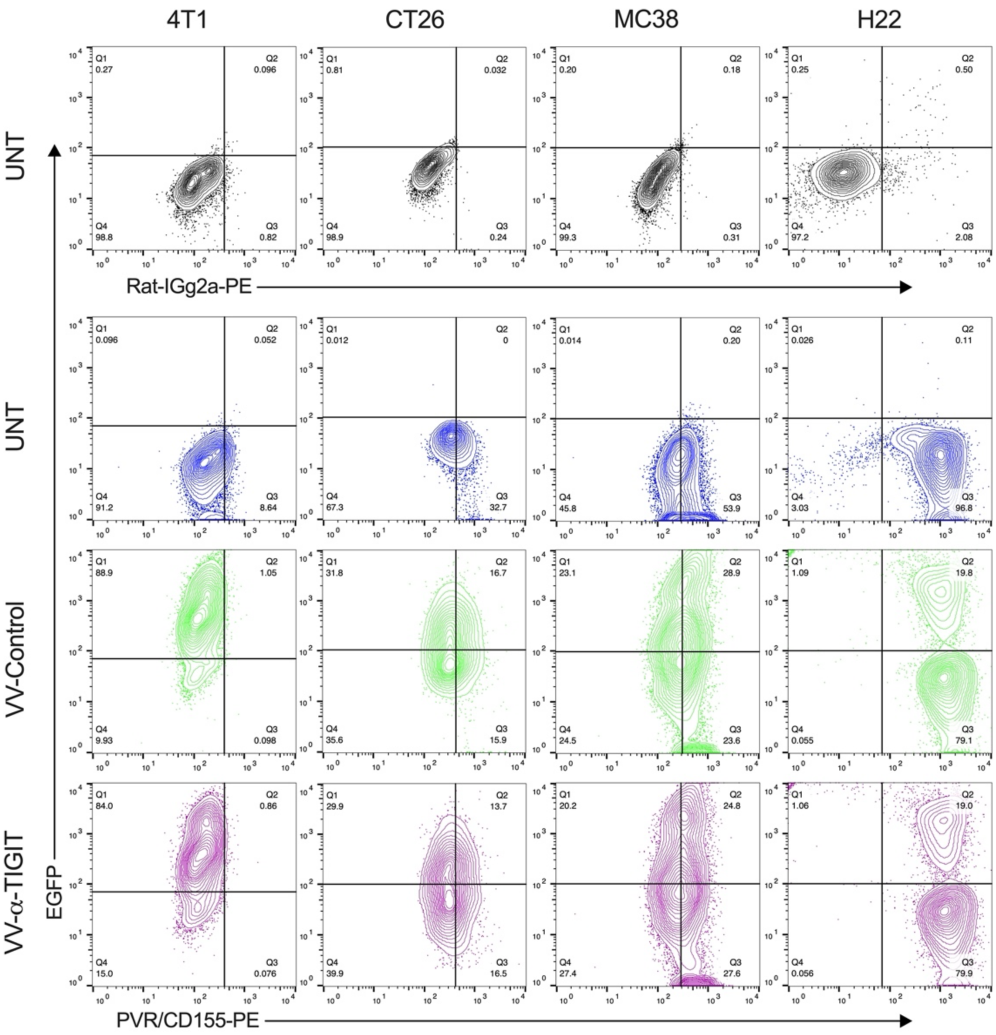

Fig. S4. Expression of PVR/CD155 on cancer cells following VV infection. 4T1, CT26, MC38, and H22 cells were infected with VV-control or VV-α-TIGIT for 48 hours at a multiplicity of infection (MOI) of 1. After that, the cells were harvested and PVR/CD155 expression on these cells was detected by flow cytometry using a PE-conjugated anti-CD155 antibody. ISO-PE, PE-conjugated isotype control antibody (Rat-anti-mouse IgG2a); UNT, untreated cells.

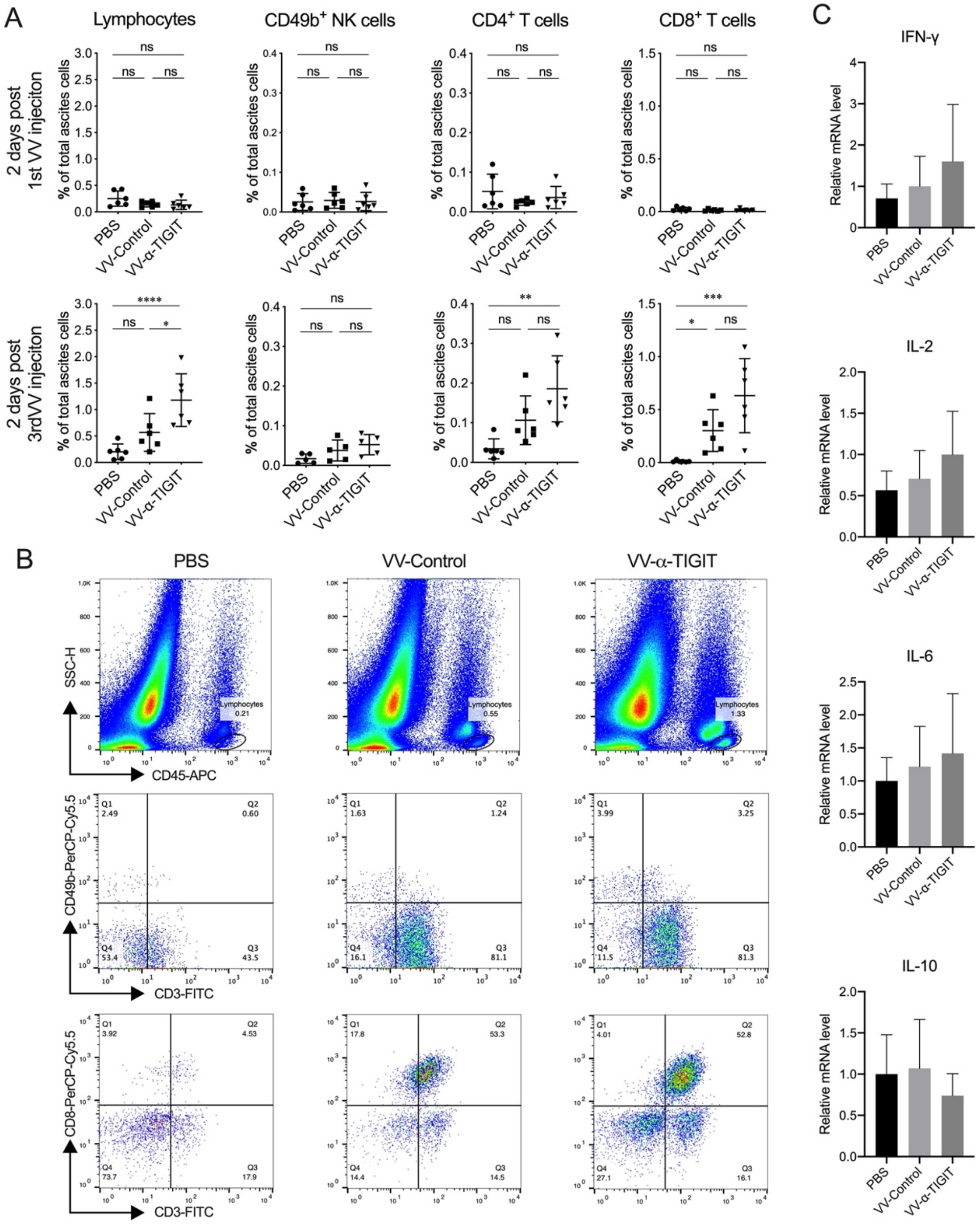

Fig. S5. Immune-cell infiltration and activation induced by VV-α-TIGIT. The ascites tumor model was established by intraperitoneal inoculation of 5×10^6 H22 cells in BALB/c mice. After ascites formation, the mice were injected intraperitoneally with 100 μL of PBS, VV-Control, or VV-α-TIGIT ($1\text{x}10^7$ PFU per mouse) for three times at a 2-day interval. The Treatment scheme was similar to **Fig. 5. A**. Two days after the first or third VV administration, ascites was harvested and tumor-infiltrating lymphocytes were analyzed by flow cytometry. B. Representative flow cytometry plots of lymphocytes and their subsets. C. Ascites cells were harvested 2 days post third VV injection and quantitative polymerase chain reaction (qPCR) was performed to detect the mRNA levels of IFN-γ, IL-2, IL-6, and IL-10. Data are presented as the mean ± SD of 7 mice for all groups.

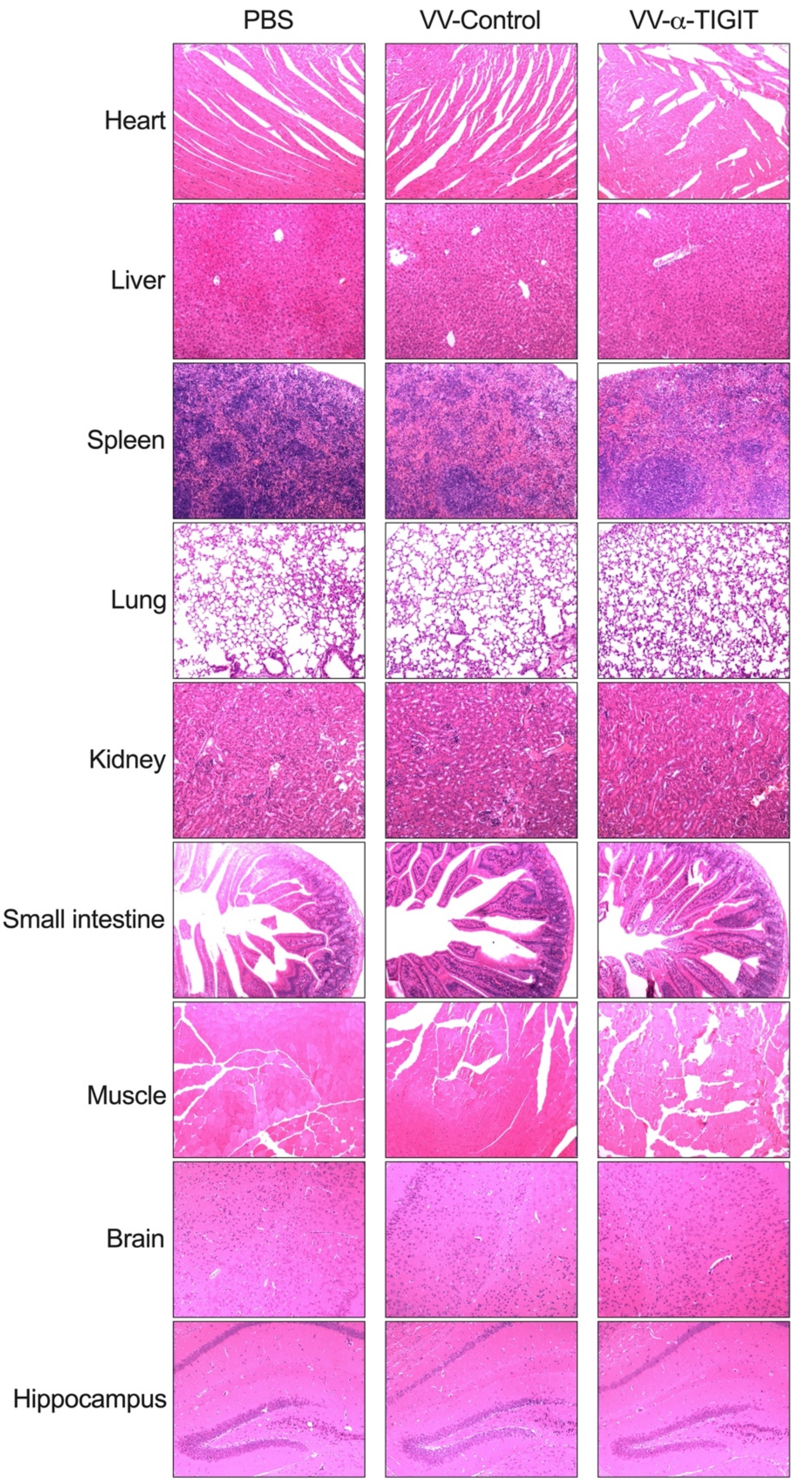

Fig. S6. Hematoxylin and eosin (H&E) staining of the tissues from heart, liver, spleen, lung, kidney, small intestine, muscle, and brain. The subcutaneous tumor model was established by inoculation of 5×10^5 CT26 cells on the right flank of BALB/c mice. When the tumor reached approximately 50 mm^3, mice were treated i.t. with PBS, 1×10^7 PFU of VV-Control or VV-α-TIGIT. Seven days after VV injection, mice was sacrificed and tissues were collected from the mice. After that, a standard H&E stanning was performed.
